# Beyond Chronological Age: A Scoping Review of Neurosurgical Intervention for Central Nervous System Tumors in the Very Elderly

**DOI:** 10.3390/curroncol33090550

**Published:** 2026-09-10

**Authors:** Jacob Gould, Sarah Segal, Garin G. Griffith, Saud K. Zaidan, Saarang Patel, Arjit Singh, Hazem S. Ghaith, Julian Gendreau

**Affiliations:** 1 Department of Neurological Surgery, NYU Grossman School of Medicine, New York, NY 10016, USA; jacob.gould@nyulangone.org; 2 Department of Neurosurgery, University of Pittsburgh School of Medicine, Pittsburgh, PA 15261, USA; 3 Department of Neurological Surgery, Oregon Health & Science University, Portland, OR 97239, USA; 4 Department of Biological Sciences, Seton Hall University, South Orange, NJ 07079, USA; 5 Department of Neuroscience, Johns Hopkins University, Baltimore, MD 21218, USA

**Keywords:** brain tumor, frailty, geriatric oncology, neuro-oncology, spinal tumor, surgical outcomes

## Abstract

Adults aged 75 years or older are increasingly considered for neurosurgical treatment of primary central nervous system tumors, yet the evidence guiding treatment selection remains fragmented. We conducted a scoping review of PubMed/MEDLINE, Ovid Embase, Scopus, and Web of Science from database inception through July 2026. Original clinical reports were eligible when they included separately extractable data for adults aged 75 years or older undergoing or being evaluated for tumor-directed neurosurgical intervention for primary intracranial, skull-base, cranial-nerve, spinal cord, meningeal, or primary vertebral tumors. Data were charted using a standardized framework and synthesized descriptively by tumor type, treatment intent, patient-selection factors, and reported outcomes. Eighty reports were included, of which 37 addressed glioma or glioblastoma and 25 addressed intracranial meningioma; 75 were retrospective. In glioma, greater resection was frequently associated with longer survival, although findings for subtotal resection were inconsistent and postoperative treatment completion was an important prognostic factor. In meningioma, functional preservation and independence were prominent considerations, while sellar, spinal, and vestibular schwannoma studies emphasized vision, neurologic function, and cranial-nerve outcomes. Quality of life, cognition, caregiver burden, and time at home were rarely reported. Surgical benefit in very elderly patients is tumor-specific, and future studies should prioritize prospective, patient-centered outcomes rather than relying primarily on survival or chronological age.

## Simple Summary

Adults aged 75 years or older are increasingly undergoing surgery for primary tumors of the brain, skull base, and spine, but it remains unclear which outcomes best define meaningful benefit in this population. We reviewed 80 published reports examining surgical outcomes, patient-selection factors, and the types of outcomes that have been studied. Most evidence was retrospective and concentrated on glioma and meningioma. The potential value of surgery differed by tumor type: survival and the ability to complete additional treatment were particularly relevant in glioma, whereas preservation of independence, vision, walking ability, or cranial-nerve function were often more relevant for other tumors. Quality of life, cognition, caregiver burden, and time at home were rarely measured. Future studies should incorporate tumor-specific, patient-centered outcomes and evaluate surgical candidacy using functional and physiologic factors rather than chronological age alone.

## 1. Introduction

Population aging correlates with an increasing number of very elderly adults diagnosed with primary tumors of the brain, meninges, skull base, cranial nerves, spinal cord, and vertebral column. Neurosurgeons are therefore increasingly asked to consider biopsy, resection, decompression, or other tumor-directed procedures in patients aged 75 years and older [1,2,3,4,5]. These decisions are particularly consequential because the potential benefits of surgery must be balanced against perioperative complications, prolonged recovery, functional decline, non-home discharge, and competing risks of mortality [6,7,8,9].

Chronological age alone is an incomplete measure of operative risk or potential benefit. Patients of the same age may differ substantially in frailty, comorbidity burden, cognition, performance status, baseline independence, and physiologic reserve [8,9,10,11]. The goals of treatment also vary by tumor type. For aggressive malignancies such as glioblastoma, surgery may provide diagnostic clarification, cytoreduction, and access to subsequent oncologic therapy [12,13,14]. For more indolent tumors, including meningiomas, pituitary adenomas, schwannomas, and spinal meningeal tumors, symptom relief and preservation of neurologic function or independence may be more important than an incremental survival benefit [3,15,16].

Despite the increasing clinical relevance of this population, adults older than 75 years remain underrepresented in prospective oncologic and surgical research. Available evidence is largely derived from retrospective institutional series, administrative databases, and cancer registries [9,17,18,19,20]. These studies vary considerably in their definitions of “elderly” and “very elderly,” tumor types, treatment comparisons, and reported outcomes. Large database studies can characterize treatment patterns and survival but often lack information on frailty, cognition, tumor anatomy, functional recovery, and patient preferences [20,21,22]. Conversely, institutional series may provide greater clinical detail but are typically small and highly selected [3,7,16].

Interpretation is further complicated by confounding by indication. Patients selected for surgery may have better baseline function, fewer comorbidities, more favorable tumor anatomy, and a greater likelihood of receiving adjuvant therapy than patients managed nonsurgically [12,13,20,21]. Associations between surgery and improved survival, therefore, cannot be assumed to represent causal treatment effects. Moreover, the literature has emphasized mortality and overall survival more consistently than outcomes such as neurologic function, discharge disposition, symptom relief, cognition, quality of life, and preservation of independence [22,23,24].

The primary objective of this review was to characterize the utilization, perioperative safety, survival outcomes, functional outcomes, and patient-selection considerations associated with neurosurgical intervention for primary intracranial, skull-base, spinal cord, spinal meningeal, and primary vertebral tumors among adults aged 75 years and older. Secondary objectives were to compare how the potential benefit of surgery is defined across tumor types, assess the alignment between reported outcomes and tumor-specific treatment goals, examine the incorporation of frailty and functional measures, and identify priorities for future patient-centered research.

## 2. Methods

### 2.1. Review Design and Ethics

This scoping review was conducted using a methodology informed by the Joanna Briggs Institute framework for scoping reviews and is reported in accordance with the Preferred Reporting Items for Systematic Reviews and Meta-Analyses Extension for Scoping Reviews (PRISMA-ScR). A scoping review design was selected because substantial heterogeneity was anticipated in tumor type, age threshold, neurosurgical intervention, treatment intent, study design, comparator, and reported outcome. The objective was therefore to map and characterize the available evidence rather than to estimate a single pooled treatment effect.

A formal review protocol was documented on the Open Science Framework under registration DOI (10.17605/OSF.IO/UWKBA). Decisions made during full-text reconciliation and evidence synthesis were documented in a structured master database. Because the review used only previously published, nonidentifiable aggregate data and involved no interaction with human participants, institutional review board approval and informed consent were not required.

### 2.2. Eligibility Criteria

Reports were eligible if they presented original clinical data involving adults aged 75 years or older who underwent or were evaluated for tumor-directed neurosurgical intervention. Studies enrolling broader age ranges were eligible only when outcomes for patients aged 75 years or older were separately extractable. The threshold of 75 years was selected a priori as a pragmatic operational definition of the very elderly population, distinguishing the target cohort from broader “elderly” populations commonly defined at younger age thresholds while retaining sufficient literature for disease-specific synthesis. Studies using higher thresholds, such as 80 or 85 years, were eligible when outcomes remained separately extractable, allowing the review to capture heterogeneity in definitions of “very elderly” without combining the target population with younger elderly cohorts. Case series were required to include at least five eligible patients to exclude isolated anecdotal reports while retaining small clinical series that are common in the literature on uncommon tumors and very elderly populations.

Eligible conditions included primary benign and malignant tumors of the brain, meninges, ventricular system, sellar or suprasellar region, skull base, cranial nerves, spinal cord, spinal meninges, and primary vertebral column. Reports limited to intracranial or spinal metastases were excluded. Mixed-tumor cohorts were eligible only when outcomes for patients with eligible primary tumors were separately extractable from metastatic tumors, non-neoplastic lesions, or other ineligible conditions.

Eligible neurosurgical interventions included biopsy, tumor resection, craniotomy, transsphenoidal surgery, endoscopic endonasal surgery, spinal tumor resection, tumor-directed decompression, and related operative procedures. Reports comparing surgery with biopsy, observation, radiotherapy, systemic therapy, or supportive management were eligible when they otherwise met population and disease criteria.

Randomized studies, prospective and retrospective cohort studies, case–control studies, institutional clinical series, registry analyses, administrative-database studies, and case series containing at least five eligible patients were considered. Case reports, case series containing fewer than five eligible patients, reviews, meta-analyses, editorials, commentaries, and reports without original clinical outcomes were excluded. Conference abstracts or other abbreviated reports were eligible only when sufficient information was available to confirm eligibility and extract at least one relevant clinical outcome. No publication date or language restrictions were applied during database searching.

### 2.3. Search Strategy

PubMed/MEDLINE, Ovid Embase, Scopus, and Web of Science Core Collection were searched from database inception through July 2026. PubMed was searched on 24 July 2026, and Ovid Embase, Scopus, and Web of Science were searched on 25 July 2026.

Search strategies combined terms representing three concepts: very elderly populations, primary central nervous system tumors, and tumor-directed neurosurgical intervention. No date, language, subject-area, or open-access restrictions were applied. Complete database-specific search strings, platforms, search fields, dates, and retrieval counts are provided in Supplementary Table S1.

### 2.4. Record Management and Study Selection

All database exports were combined in a structured master database while preserving the originating database and source-record identifiers. Deduplication used a combination of PubMed identifiers, digital object identifiers, normalized titles, authors, publication years, journal information, and publication type. Digital object identifiers were not used in isolation because conference supplements and related records could share nonunique or erroneous metadata.

Potential duplicate records with conflicting identifiers, variant titles, conference-to-full-publication relationships, or discrepant DOI information were manually reviewed. When duplicate publications were confirmed, a single canonical report-level identifier was retained. The unit of screening and inclusion was the published report rather than the presumed underlying patient cohort because multiple reports could arise from the same or an overlapping data source.

Two reviewers independently screened titles and abstracts and classified each record as potentially eligible or ineligible. Reports considered potentially eligible underwent full-text retrieval, and the same two reviewers independently assessed each full-text report against the prespecified eligibility criteria. Disagreements at either stage were resolved through discussion and, when consensus could not be reached, adjudication by an additional reviewer.

A single primary exclusion reason was assigned to each report excluded during eligibility review. Particular attention was given to mixed-tumor reports to determine whether outcomes for eligible primary tumors could be separated from metastatic disease or non-neoplastic conditions. Reports in which eligible primary-tumor outcomes were inseparable from ineligible conditions were excluded during the final eligibility reconciliation.

The study-selection process was documented at each stage of identification, screening, retrieval, eligibility assessment, and inclusion and is summarized in the PRISMA flow diagram. 

### 2.5. Data Charting

A standardized data-charting framework was developed before formal extraction and refined as tumor-specific outcome domains became apparent during the review. One reviewer performed primary data extraction for all included reports. Key study characteristics, patient-selection variables, and quantitative outcomes used in the synthesis were independently verified by a second reviewer. Discrepancies identified during verification were resolved through review of the original report and consensus. Study investigators were not contacted to obtain additional or unpublished data; data charting was based on information available in the retrieved reports.

Extracted report-level variables included publication year, country or setting, study design, institutional or database source, study period, age threshold, sample size, age-eligible sample size, tumor diagnosis, tumor grade or molecular characteristics when reported, neurosurgical intervention, treatment comparator, extent of resection, adjuvant treatment, follow-up, and relevant patient-selection measures.

Clinical variables included performance status, frailty, comorbidity, ASA classification, cognition, baseline independence, tumor size and location, edema, neurologic status, and other reported selection factors. Outcome domains included perioperative complications, early mortality, length of stay, readmission, discharge disposition, rehabilitation, neurologic function, KPS, mRS, ambulation, visual function, endocrine function, cranial-nerve outcomes, symptom relief, treatment receipt or completion, local control, recurrence, progression-free survival, and overall survival.

Resection terminology was standardized throughout the review. Gross-total resection (GTR) denotes source-reported complete resection, subtotal resection (STR) denotes source-reported incomplete cytoreductive resection, and biopsy denotes diagnostic tissue sampling without intended cytoreduction. The term maximal resection was retained when explicitly used by the source, whereas “maximal safe resection” is used in the present review as a clinical principle emphasizing the greatest achievable cytoreduction without unacceptable neurologic injury.

Adjusted and unadjusted estimates were charted separately. When available, multivariable hazard ratios, odds ratios, confidence intervals, and *p*-values were preferentially used for comparative interpretation. Effect estimates were retained as reported and were not transformed into a common effect measure.

Formal study-level critical appraisal or risk-of-bias assessment was not undertaken because the objective of this scoping review was to map the breadth, characteristics, and outcome reporting of a heterogeneous evidence base rather than to estimate a pooled treatment effect or formally grade the certainty of evidence.

### 2.6. Cohort Overlap and Evidence-Source Assessment

Because multiple included reports analyzed national databases or potentially overlapping institutional cohorts, an explicit overlap assessment was performed. Reports were compared using the named data source or institution, tumor diagnosis, study period, eligibility criteria, patient age, sample size, author group, and outcome domain.

Relationships between reports were classified as confirmed identical, confirmed nested, likely overlapping, potentially overlapping, or apparently distinct when sufficient information was available. Reports from the same database family were retained when they addressed distinct clinical questions or provided unique age-specific outcomes, but concordant findings were not interpreted as independent patient-level replication.

Database-family reuse was summarized separately from report count. Report-level denominators were therefore used throughout the scoping review unless a distinct underlying cohort could be established with confidence. When a single publication contained analytically distinct populations from different data sources, these were treated as separate evidence units for the relevant tumor-specific analysis while retaining the publication as a single included report.

### 2.7. Evidence Classification and Tumor-Specific Synthesis

Included reports were first categorized by tumor group: glioma or glioblastoma, intracranial meningioma, sellar or suprasellar tumors, mixed or other primary intracranial tumors, spinal or primary vertebral tumors, and vestibular schwannoma.

Within each tumor category, evidence was organized according to the clinical question most relevant to treatment intent rather than forcing a common cross-tumor endpoint. Glioma analyses emphasized the extent of resection, survival, postoperative functional preservation, patient selection, receipt of adjuvant therapy, and molecular treatment modifiers. Meningioma analyses separately evaluated perioperative safety, functional independence, and histology-specific oncologic management. Sellar analyses emphasized visual recovery, endocrine outcomes, complications, resection strategy, and tumor control. Spinal analyses focused on neurologic recovery, ambulation, perioperative risk, and survival. Vestibular schwannoma analyses emphasized facial-nerve function, hearing, balance, disposition, independence, complications, and residual-tumor control.

Reports were considered primary age-specific evidence only when results for patients aged 75 years or older were directly extractable. Findings reported only for broader elderly populations were retained as contextual evidence when relevant but were not treated as direct evidence for the target population.

### 2.8. Descriptive Aggregation

Formal meta-analysis was not performed because of substantial heterogeneity in tumor biology, treatment intent, age definition, intervention, comparator, outcome definition, follow-up, and analytic methodology.

Where multiple reports used directly comparable outcome definitions and provided complete numerators and denominators, raw event counts were occasionally combined descriptively to illustrate the direction and availability of evidence. Such calculations were explicitly treated as descriptive aggregations rather than pooled effect estimates; no inverse-variance weighting, between-study modeling, pooled confidence intervals, or tests of heterogeneity were performed.

Study-level ranges, counts, proportions, adjusted estimates, and survival measures were otherwise presented without statistical pooling. No vote-counting procedure was used to infer treatment effectiveness from the number of statistically significant reports.

### 2.9. Evidence-Base Architecture

The overall structure of the literature was characterized using report counts and proportions by tumor category, publication period, study design, and data-source architecture. Clinical cohorts were distinguished from population registries and national administrative or clinical databases. Multicenter representation and repeated use of major database families were also quantified.

Because the literature contained confirmed and likely overlapping populations, the number of included reports was not interpreted as the number of independent patient cohorts.

### 2.10. Outcome-Reporting Evidence Map

To evaluate whether the outcomes reported in the literature aligned with clinically meaningful treatment goals, an outcome-reporting evidence map was constructed across tumor categories. Eight domains were assessed: overall survival; function or independence; complications or early mortality; local control or progression-free survival; quality of life; cognition; treatment completion or pathway feasibility; and time at home or caregiver burden.

Reporting availability was categorized using an ordinal descriptive scale: 0 indicated that the domain was absent; 1 indicated limited, indirect, or incomplete reporting; 2 indicated direct reporting in at least one report; and 3 indicated direct reporting in multiple reports. The score represented the availability of reporting only and was not intended to measure study quality, strength of evidence, or treatment effectiveness. The resulting matrix was used to identify areas of evidence concentration and persistent patient-centered outcome gaps.

### 2.11. Cross-Tumor Evidence Synthesis

After completion of the tumor-specific analyses, recurrent themes were examined across disease categories. Particular attention was given to the role of chronological age, baseline functional and physiologic reserve, achievable surgical benefit, postoperative neurologic preservation, feasibility of adjuvant or salvage treatment, availability of lower-burden alternatives, and the relationship between treatment burden and remaining-life priorities.

These recurring themes were organized into an evidence-informed cross-tumor oncogeriatric framework. The framework was developed as a structured synthesis of the included observational literature rather than as a validated clinical prediction rule. It was not used to assign treatment recommendations to individual patients or to generate quantitative estimates of net benefit.

### 2.12. Statistical Analysis

Categorical characteristics were summarized using counts and percentages. Continuous variables were summarized using medians, ranges, or study-level values when sufficiently comparable data were available. Percentages were calculated using the relevant report-level or analysis-specific denominator rather than the total corpus when only a subset of reports contributed to an analysis.

No meta-analysis, meta-regression, pooled survival analysis, or formal comparative-effect modeling was performed. *p*-values, confidence intervals, hazard ratios, and odds ratios presented in the review were those reported by the original investigators. Observational associations between surgery, extent of resection, adjuvant treatment, and outcomes were interpreted as associations and not as causal treatment effects.

## 3. Results

### 3.1. Study Selection

**Figure 1:**
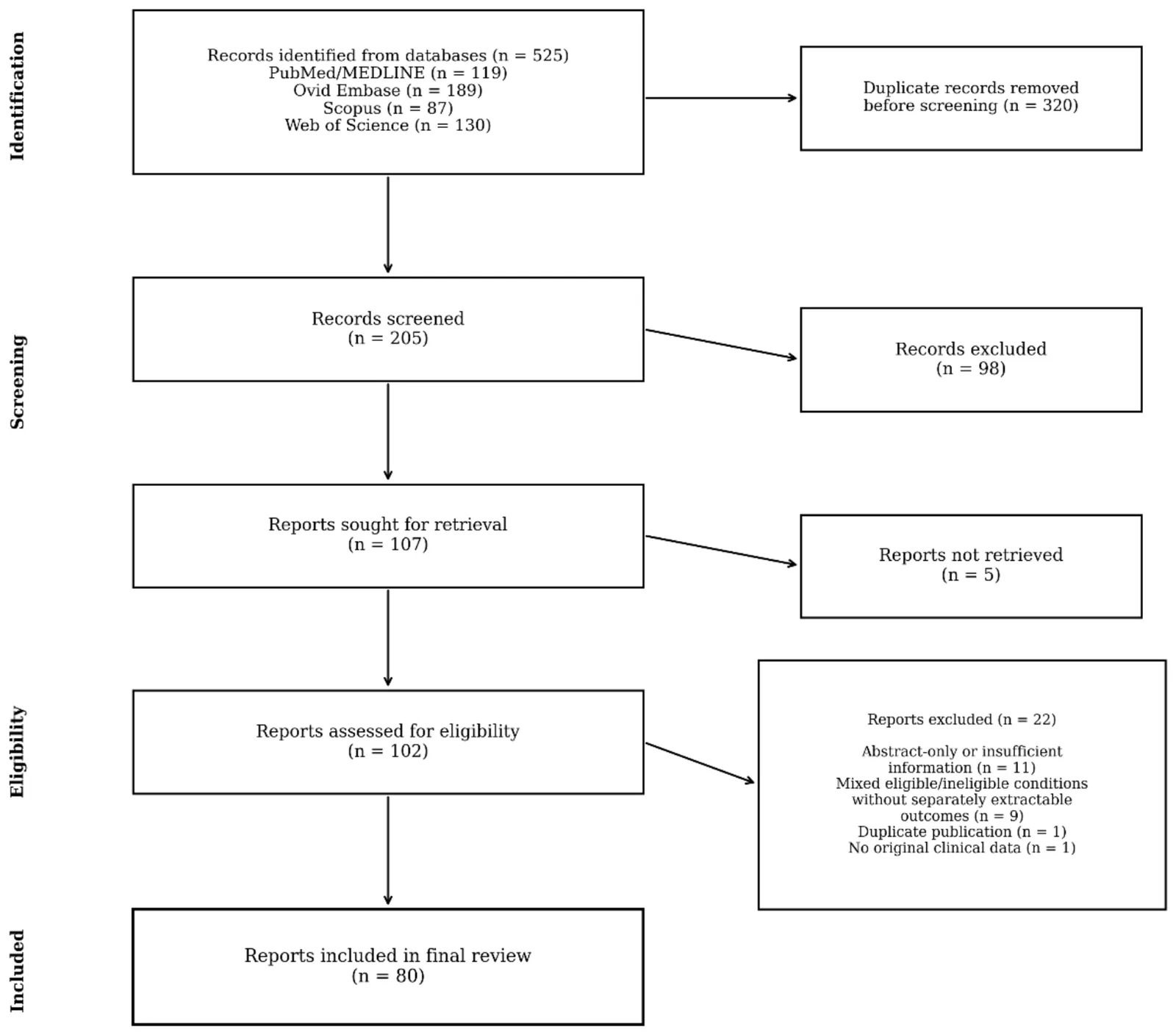
PRISMA flow diagram of study selection.

The database searches identified 525 records, including 119 from PubMed/MEDLINE, 189 from Ovid Embase, 87 from Scopus, and 130 from Web of Science. After the removal of 320 duplicate records, 205 unique records underwent title and abstract screening. Ninety-eight records were excluded at this stage, leaving 107 reports sought for retrieval. Five reports could not be retrieved, and 102 full-text reports were assessed for eligibility. Twenty-two reports were excluded following full-text review: 11 were available only as abstracts or otherwise contained insufficient information to confirm eligibility and extract relevant outcomes, nine combined eligible primary tumors with central nervous system metastases or non-neoplastic conditions without separately extractable primary-tumor outcomes, one was a duplicate publication, and one contained no original clinical data. Eighty reports were included in the final review (Figure 1).

### 3.2. Characteristics of the Evidence Base

Glioma and glioblastoma were the most frequently studied tumors, accounting for 37 of 80 reports (46.3%), followed by intracranial meningioma in 25 reports (31.3%). Sellar or suprasellar tumors were evaluated in eight reports (10.0%), other primary intracranial tumors in five (6.3%), spinal or primary axial tumors in three (3.8%), and vestibular schwannoma in two (2.5%). Glioma and meningioma together accounted for 62 reports (77.5%) (Table 1).

The literature was concentrated in recent years. Seven reports (8.8%) were published from 1995 through 2009, three (3.8%) from 2010 through 2014, 19 (23.8%) from 2015 through 2019, 38 (47.5%) from 2020 through 2024, and 13 (16.3%) from 2025 through July 2026. Overall, 70 reports (87.5%) were published in 2015 or later, including 51 (63.8%) published from 2020 through July 2026. Thus, the evidence base was weighted strongly toward contemporary practice. Formal comparison of outcomes across publication eras was not undertaken because differences in tumor classification, case selection, adjuvant therapy, study design, and outcome reporting would substantially confound a temporal treatment effect.

The evidence base was predominantly retrospective. Forty-six reports (57.5%) described retrospective clinical cohorts or case series, and 29 (36.3%) were retrospective database or registry analyses. Two reports used mixed prospective–retrospective cohorts, one retrospectively analyzed a prospectively maintained database, one described a prospective observational cohort, and one reported a prospective single-arm phase II study. Thus, 75 of 80 reports (93.8%) were purely retrospective, only two reports described prospectively conducted studies, and no randomized treatment-comparison study was identified.

Fifty-one reports (63.8%) were based on institutional or multicenter clinical cohorts, whereas 29 (36.3%) used population registries, claims data, or national administrative databases. Only seven of the 51 clinical-cohort reports (13.7%) were explicitly multicenter. Among the 29 database reports, 27 (93.1%) arose from repeatedly used source families. Seventeen used SEER or SEER-Medicare, seven used the National Cancer Database, two used the Japanese Diagnosis Procedure Combination database, and one each used ACS-NSQIP, the Korean National Health Insurance Service, and CBTRUS. Confirmed or likely overlap was identified within several SEER, NCDB, and Japanese database clusters; consequently, concordant findings from these reports were not treated as independent patient-level replication.

Characteristics of each included report, including study design, data source, study period, age criterion, age-eligible sample size, tumor type, neurosurgical intervention, comparator when applicable, and follow-up, are provided in Supplementary Table S2.

### 3.3. Patient-Level Frailty and Functional Reserve

Patient-level reserve was incompletely characterized across the literature. Among 20 glioma reports providing directly applicable evidence regarding patient selection or the postoperative treatment pathway, 17 (85.0%) reported performance or functional measures, whereas six (30.0%) reported comorbidity or frailty and only one (5.0%) evaluated sarcopenia or body composition. Among 18 age-specific meningioma surgical reports, 14 (77.8%) reported function, independence, or disposition, while only three (16.7%) incorporated a formal frailty measure. Across tumor categories, direct measures such as KPS, ECOG, mRS, ASA class, comorbidity burden, and baseline independence were more consistently represented than formal frailty assessment, while cognition, nutritional reserve, sarcopenia, and social support were rarely evaluated. No included report validated a tumor-agnostic frailty threshold for surgical candidacy.

### 3.4. Evolution of Neurosurgical Care and Historical Applicability

The risk–benefit profile of neurosurgery in older adults has evolved alongside advances in neuroimaging, operative visualization and navigation, intraoperative monitoring, anesthesia, critical care, minimally invasive techniques, and perioperative management. Chibbaro et al., examining patients aged 70 years or older over a 25-year period at a major academic neurosurgical center, demonstrated increasing operative treatment of elderly patients together with changing procedural practice and declining mortality, illustrating why historical estimates of surgical risk may not directly represent contemporary practice [25]. The present review is predominantly contemporary, with 87.5% of included reports published since 2015. Nevertheless, the publication era cannot isolate the effects of technological progress because contemporary cohorts also differ in patient selection, molecular classification, adjuvant therapy, and outcome ascertainment. Historical studies therefore remain informative, but their absolute morbidity and mortality estimates should be applied cautiously to current practice.

### 3.5. Glioma and Glioblastoma

Thirty-seven reports evaluated glioma or glioblastoma [1,6,10,12,13,14,17,18,21,22,26,27,28,29,30,31,32,33,34,35,36,37,38,39,40,41,42,43,44,45,46,47,48,49,50,51,52]. Twelve reports provided an age-specific comparison of surgical strategies, yielding 13 evidence units because one report included separate institutional and SEER cohorts (Table 2, Panel A). Five evidence units were derived from population registries, and all five reported an adjusted association between more extensive resection and longer overall survival. In contrast, only four of eight institutional evidence units demonstrated an adjusted survival association. Seven of the 13 evidence units reported perioperative or functional outcomes, and no randomized or prospective controlled surgical comparison was identified.

Across registry analyses, gross-total or maximal resection was more consistently associated with longer survival than subtotal resection. In a SEER analysis of patients aged 80 years or older, gross-total resection was associated with lower mortality than biopsy (hazard ratio [HR], 0.696; 95% confidence interval [CI], 0.619–0.781), whereas subtotal resection was not (HR, 0.997; 95% CI, 0.890–1.117) [21]. In another SEER cohort of patients aged 75 years or older, median overall survival was two months without surgery, three months after biopsy, four months after subtotal or partial resection, and six months after gross-total resection. Relative to biopsy, the adjusted HR was 0.796 (95% CI, 0.665–0.951) for subtotal or partial resection and 0.566 for gross-total resection [13]. A separate analysis of patients aged 80 years or older reported median overall survival of two months without surgery, three months after biopsy or partial resection, three months after subtotal resection, and five months after gross-total resection [41].

The clinically detailed institutional evidence was less uniform. In one cohort of 85 patients aged 75–84 years, gross-total resection was associated with an adjusted survival increase of 3.90 months compared with biopsy (95% CI, 1.47–6.33; *p* = 0.002), whereas subtotal resection was not associated with longer survival. Both gross-total and subtotal resection were associated with greater postoperative Karnofsky Performance Status decline than biopsy [12]. In a cohort of 108 patients aged 75 years or older, maximal resection was associated with lower mortality than submaximal resection (HR, 0.379; 95% CI, 0.218–0.659), whereas biopsy was not independently different from submaximal resection [50]. In a lobar glioblastoma subgroup aged 80 years or older, resection was associated with longer survival than biopsy after adjustment for MGMT status and adjuvant treatment (HR, 0.28; 95% CI, 0.10–0.81) [14]. Other small institutional cohorts found no statistically significant independent survival difference between resection and biopsy [6,37,40,51].

Twenty reports provided directly applicable evidence regarding patient selection or the postoperative treatment pathway (Table 2, Panel B). Performance status or functional measures were reported in 17 of 20 reports (85.0%), adjuvant-treatment receipt in 14 (70.0%), postoperative function, discharge, or recovery in eight (40.0%), comorbidity or frailty in six (30.0%), tumor anatomy or burden in four (20.0%), MGMT-stratified treatment effects in three (15.0%), and sarcopenia or body composition in one (5.0%).

Chronological age was independently associated with survival in only one of six age-specific multivariable models that included age. Performance status was independently associated with survival in four of eight models. By contrast, every age-specific multivariable model that included postoperative radiotherapy, temozolomide, or combined chemoradiotherapy identified at least one adjuvant-treatment variable as independently associated with longer survival (9 of 9 models).

Five reports directly identified postoperative complications, functional decline, non-home discharge, or prolonged recovery as adverse treatment-pathway events. In one cohort, postoperative complications were associated with markedly higher mortality (HR, 5.43; 95% CI, 1.73–17.04), and the apparent survival gradient across extent-of-resection groups was no longer present among patients who experienced complications [6]. Another cohort found that postoperative KPS decline reduced receipt of adjuvant therapy from 77.6% to 50.0% [10]. Three age-specific studies further reported stronger treatment or resection associations among patients with MGMT-methylated tumors than among those with unmethylated tumors [14,43,50].

Overall, the glioma evidence associated gross-total or maximal resection with longer survival more consistently than subtotal resection. However, survival was most reproducibly associated with completion of postoperative radiotherapy and/or temozolomide, and the clinical value of cytoreduction varied according to tumor anatomy, postoperative functional preservation, molecular status, and the feasibility of completing multimodal therapy.

### 3.6. Intracranial Meningioma

Twenty-five reports evaluated intracranial meningioma [2,7,8,11,15,19,20,53,54,55,56,57,58,59,60,61,62,63,64,65,66,67,68,69,70]. Eighteen reports provided direct age-specific evidence regarding surgical safety, function, or patient selection, representing 17 underlying source populations because two reports analyzed the same 8138-patient Japanese administrative cohort (Table 3, Panel A). All 18 reports evaluated perioperative morbidity or mortality; 14 (77.8%) reported functional status, independence, or discharge outcomes; 10 (55.6%) evaluated at least one adjusted selection factor; three (16.7%) used a formal frailty measure; six (33.3%) identified tumor size, volume, edema, or location as a risk factor; and six (33.3%) reported functional outcomes at approximately one year.

Several selected cohorts reported functional improvement or stability after surgery. In one series, 31 of 37 patients improved or remained stable at discharge [65]. Another demonstrated significant improvement in one-year modified Rankin Scale scores [15]. In the largest multicenter supratentorial cohort, 38.5% of patients improved and 33.2% remained unchanged at one year; however, 28.2% were functionally worse, including patients who died. Surgery-associated complications occurred in 42.4%, 90-day mortality was 9.0%, and one-year mortality was 13.2% [7].

A second contemporary multicenter cohort of 189 patients aged 80 years or older reported new neurologic deficits in 23.8%, postoperative complications in 42.8%, 30-day mortality of 4.2%, and one-year mortality of 15.8%. Mean KPS declined from 73.5 before surgery to 63.5 at 12 months, and 27.1% of patients remained functionally independent at 12 months [8].

Baseline functional status and postoperative neurologic preservation were among the strongest patient-level predictors. Preoperative KPS below 70 was associated with an unfavorable composite outcome (odds ratio [OR], 3.10; 95% CI, 1.44–6.68) and with KPS below 70 at 12 months (OR, 14.45; 95% CI, 5.64–37.03). A new postoperative neurologic deficit independently predicted 12-month KPS below 70 (OR, 4.79; 95% CI, 1.59–14.38) [8]. Cardiovascular comorbidity was associated with postoperative complications in another cohort (OR, 3.94; 95% CI, 1.05–14.76), and ASA class predicted complications in a matched octogenarian analysis (OR, 2.22; 95% CI, 1.02–4.81) [62,64].

Anatomic burden also influenced outcomes. In the largest supratentorial cohort, tumor diameter of at least 5 cm was associated with postoperative complications (OR, 1.87; 95% CI, 1.12–3.13), as was large tumor volume (OR, 2.35; 95% CI, 1.01–5.50) [7]. Severe edema, critical location, and prolonged operative duration were additionally associated with adverse outcomes in smaller cohorts. Formal frailty indices were infrequently used and did not consistently outperform direct measures of function or comorbidity.

Six reports evaluated oncologic management or population-level survival, but they arose from only two repeatedly used national database families (Table 3, Panel B) [2,20,56,63,69,70]. Three reports provided direct treatment-effect estimates for patients aged 80 years or older, whereas the remaining reports contributed treatment-utilization data, age-specific survival estimates, or treatment effects pooled across broader elderly populations.

Radiotherapy findings were otherwise inconsistent. Radiation alone was not associated with longer survival than observation in the high-grade SEER cohort, while radiotherapy was strongly associated with longer survival in the mixed-histology and atypical-meningioma cohorts [20,63,70]. Radiotherapy was administered to only 5.6% and 14.1% of the relevant very elderly cohorts, respectively. None of the six oncologic reports used age-specific progression-free survival, local control, or meningioma-specific mortality as the principal treatment-effect endpoint.

Across the meningioma literature, baseline functional status, ASA or comorbidity burden, tumor size and edema, and avoidance of new postoperative neurologic deficits were generally more informative for individual selection than chronological age or formal frailty indices alone (Table 3, Panel C).

### 3.7. Sellar and Suprasellar Tumors

Eight reports evaluated sellar or suprasellar tumors, including six institutional pituitary surgical cohorts comprising 242 patients aged 75 years or older and two SEER survival analyses (Table 4, Panel A) [3,23,24,71,72,73,74,75]. Five reports assessed visual outcomes, six assessed endocrine outcomes, and five reported tumor-control follow-up. No prospective comparative or randomized study was identified. Visual recovery was the most consistently reported benefit of pituitary surgery. Four cohorts provided complete improved, stable, and worsened visual-outcome categories. Across these cohorts, 113 of 158 assessed patients improved, 43 remained stable, and two worsened. This was a descriptive aggregation rather than a pooled estimate. A fifth cohort reported visual worsening in three of 16 assessed octogenarians (18.8%), significantly more frequently than among patients aged 70–79 years [74].

The largest contemporary institutional cohort included 155 patients aged 75 years or older. Among 123 patients with preoperative visual deficits, 82 improved and 41 remained stable, with no postoperative visual worsening. All 125 compressed visual pathways were radiographically decompressed. Immediate surgical complications occurred in 5.0%, 30-day mortality was 0.6%, postoperative cerebrospinal fluid leakage occurred in 1.9%, and single-surgery disease control was achieved in 97.0% [3].

Endocrine recovery was less frequent than visual improvement. One cohort found no meaningful recovery of pre-existing pituitary deficits [71]. In another, one of four patients with partial anterior pituitary deficits improved, but none normalized [73]. In the largest cohort, normal pituitary function was restored in 10 of 95 patients with preoperative deficiency (11.0%). New anterior pituitary deficits developed in three of 60 patients with normal preoperative function (5.0%) [3]. Persistent diabetes insipidus was not reported in the five studies that specifically evaluated it, although transient diabetes insipidus, syndrome of inappropriate antidiuretic hormone secretion, hyponatremia, and other electrolyte disturbances occurred [3,71,72,73,74].

Visual decompression and durable tumor control were frequently achieved without gross-total resection. One cohort reported visual improvement in 91.7% despite total removal in only 16.6% [72]. In the largest cohort, complete resection was achieved in 22%, but single-surgery disease control was 97.0%; estimated disease control was 97.3% at two years and 86.2% at five years. Extended endonasal transtuberculum surgery was independently associated with immediate complications (OR, 13.77; 95% CI, 1.77–107.17; *p* = 0.012) [3]. The two registry studies produced opposing survival associations for subtotal resection in pituitary adenoma and craniopharyngioma and did not contain visual, endocrine, functional, or perioperative outcomes [23,24].

### 3.8. Other Primary Intracranial Tumors

After full-text eligibility correction, five reports remained in the mixed/other primary intracranial category [77,78,79,80,81]. Four provided clinical outcomes, and one contributed descriptive neuropathologic data. The retained reports were too heterogeneous for a unified treatment-effect analysis.

Diagnosis-specific data demonstrated substantial variation. In one octogenarian craniotomy cohort, patients with meningioma had a 30-day readmission rate of 3.3%, 30-day mortality of 6.7%, three-month mortality of 10.0%, and mean survival of 33 months. Patients with glioblastoma from the same cohort had a 30-day readmission rate of 26.7%, 30-day mortality of 6.7%, three-month mortality of 33.3%, and mean survival of 6.9 months [81].

A historical cohort of 18 patients older than 80 years with benign primary tumors reported improvement in six, unchanged status in eight, deterioration in one, and death in three. Among 10 patients with primary malignant tumors, four improved, four remained unchanged, two worsened, and none died during the reported perioperative period [77]. Another historical series of 11 patients with rare benign primary tumors reported a good outcome in six, unchanged status in one, and death in four.

A national cohort of 130 patients aged 80 years or older with unspecified primary malignant brain tumors reported 12-month overall survival of 48.4% without postoperative treatment, 63.0% with radiotherapy, 50.0% with chemotherapy, and 69.7% with chemoradiotherapy. Treatment modality was not significantly associated with survival within the age-eligible subgroup (*p* = 0.275) [80].

### 3.9. Spinal and Primary Axial Tumors

Three reports evaluated spinal or primary axial tumors, comprising one institutional spinal meningioma cohort and registry analyses of spinal ependymoma and spinal/axial osteosarcoma (Table 4, Panel B) [4,9,76].

Among 30 patients aged 80 years or older undergoing resection of spinal meningioma, the mean motor score improved from 85.9 to 93.6 and the mean modified McCormick score improved from 3.2 to 1.8; both differences were statistically significant (*p* < 0.001). In-hospital mortality was 6.7%, 90-day mortality was 10.0%, and 30-day readmission was 10.0%. The age-adjusted Charlson Comorbidity Index independently predicted complications (OR, 2.1; 95% CI, 1.1–4.3) [4].

The spinal ependymoma analysis included 126 patients aged 75 years or older. Surgery was performed in 70% of patients aged 75 years or older compared with 85% of those aged 65–74 years (*p* < 0.001). Median overall survival was 106.0 months among surgically treated patients and 59.7 months among those who did not undergo surgery. Surgery was independently associated with lower mortality (HR, 0.46; 95% CI, 0.24–0.89), while increasing age, Charlson–Deyo score of at least 2, and increasing tumor size were associated with higher mortality. Neurologic outcomes, extent of resection, and perioperative complications were not available [9].

The spinal/axial osteosarcoma analysis included 117 patients aged 75 years or older, of whom 21 underwent subtotal resection and five underwent gross-total resection. Compared with no resection, subtotal resection was associated with lower mortality (HR, 0.499; 95% CI, 0.279–0.892), as was gross-total resection (HR, 0.563; 95% CI, 0.222–0.731). The cohort combined vertebral-column tumors with sacral or pelvic primary sites, and margin status, neurologic function, perioperative morbidity, and treatment toxicity were not reported [76].

### 3.10. Vestibular Schwannoma

Two institutional reports evaluated 37 surgically treated patients aged 75 years or older, including 21 octogenarians (Table 4, Panel C) [5,16]. Surgery was generally reserved for large, progressive, compressive, hydrocephalic, or severely symptomatic tumors after observation or stereotactic radiosurgery was considered inadequate.

Non-total resection was common. In one cohort, gross-total resection was performed in 20.8%, near-total resection in 12.5%, and subtotal resection in 66.7% [5]. In the second cohort, planned partial resection was performed in 92.3%, with gross-total resection in one of 13 patients [16].

Facial-nerve outcomes differed between cohorts. Favorable House–Brackmann grade I–II function was present at follow-up in 14 of 24 patients in one cohort, including two of seven evaluable octogenarians (28.6%) [5]. In the second cohort, favorable facial function was present in 10 of 13 patients (77%), and the facial nerve was anatomically preserved in all patients. Across both reports, only three patients had serviceable or useful ipsilateral hearing preoperatively, and none retained serviceable hearing after surgery [16].

Functional recovery was reported despite frequent rehabilitation needs. In one cohort, nine patients required out-of-home disposition, including six of eight octogenarians, but all ultimately returned to independent living [5]. In the second, severe imbalance improved in eight of 11 patients; eight were discharged home and five to rehabilitation [16].

Tumor control was achieved in 18 of 22 evaluable patients in one cohort. All four progressions occurred in octogenarians after subtotal resection [5]. In the second cohort, two recurrences followed partial resection, including one episode of rapid cystic regrowth associated with a later tumor-related death [16].

### 3.11. Cross-Tumor Outcome Reporting

**Figure 2:**
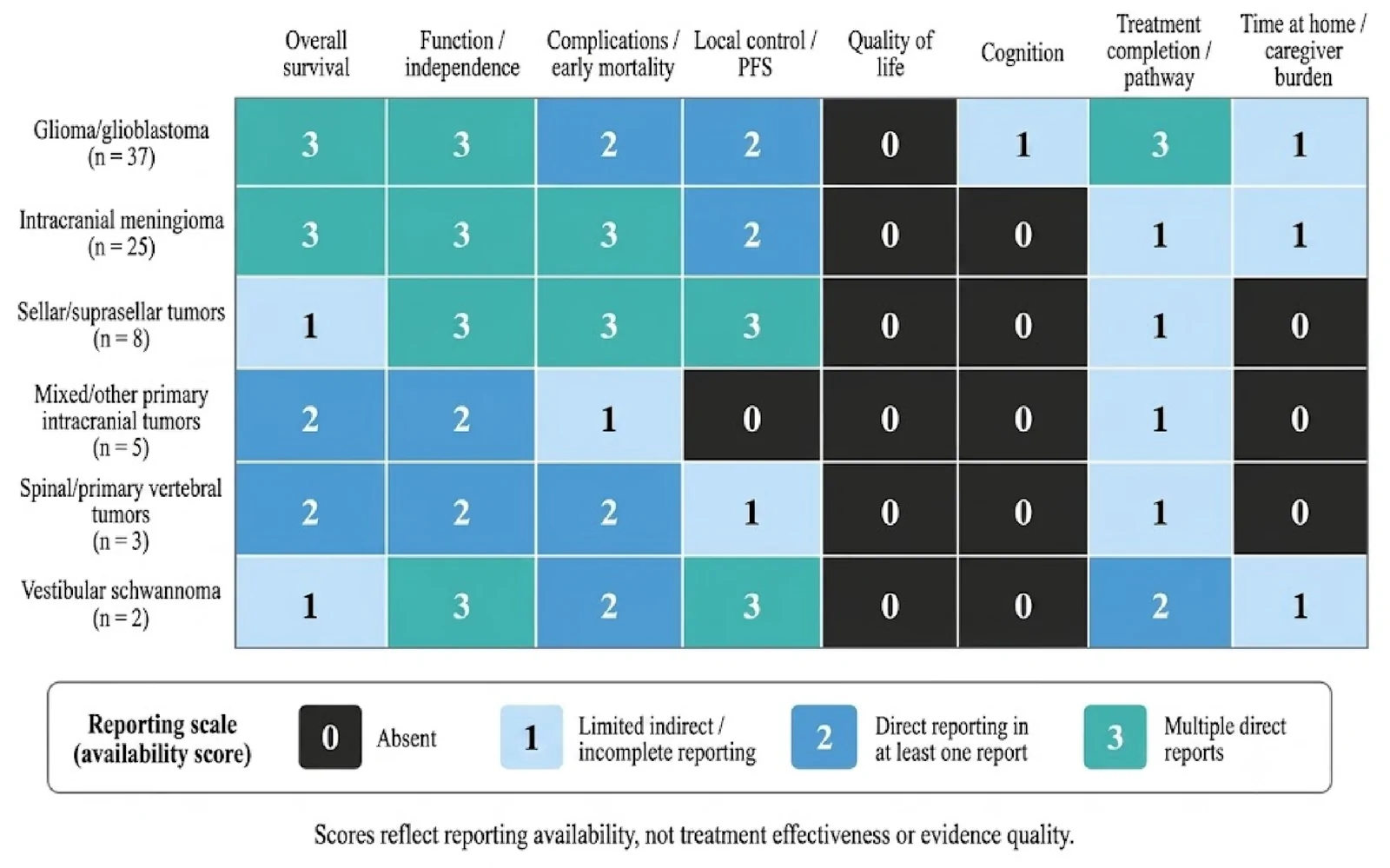
Outcome-reporting evidence map across tumor categories.

The outcome domains represented in the literature varied substantially by tumor category (Figure 2). Glioma reports emphasized overall survival and receipt of postoperative therapy; meningioma reports more frequently included functional status, complications, and independence; sellar reports focused on visual, endocrine, and tumor-control outcomes; and vestibular schwannoma reports emphasized facial function, balance, disposition, and residual-tumor control.

Patient-reported quality of life was not systematically reported in any tumor category. Cognitive outcomes were largely absent, with only limited indirect evidence in glioma. Time at home and caregiver burden were rarely measured. Sellar studies showed the closest alignment between the clinical purpose of surgery and the outcomes reported, whereas spinal and heterogeneous mixed-tumor reports frequently relied on overall survival without corresponding neurologic or patient-centered outcomes.

Across tumor categories, chronological age was inconsistently associated with outcomes after adjustment. Functional status, comorbidity, tumor anatomy, probability of neurologic preservation, and feasibility of postoperative or salvage treatment were more consistently associated with clinical outcomes. No included report validated a tumor-agnostic selection model or directly quantified the combined effects of survival, independence, treatment burden, cognition, and remaining time at home.

## 4. Discussion

This scoping review identified 80 reports, predominantly retrospective and concentrated in glioma and intracranial meningioma, with substantially less evidence for other primary central nervous system tumors. Across tumor types, the definition of meaningful surgical success differed according to disease biology and treatment intent, while cognition, quality of life, caregiver burden, and time at home were rarely measured. Because the literature was observational and repeatedly used several national database populations, the available evidence supports tumor-specific associations and decision principles rather than causal estimates of surgical benefit.

### 4.1. Frailty, Functional Reserve, and Surgical Selection

Chronological age was inconsistently associated with outcomes after adjustment within cohorts already restricted to very elderly patients. In contrast, functional status, comorbidity, physiologic reserve, tumor anatomy, neurologic trajectory, and feasibility of postoperative therapy were more consistently informative. Age therefore appears to function partly as an imprecise proxy for biologic and functional heterogeneity rather than as a sufficient measure of operative fitness.

No single frailty instrument emerged as a validated selection tool. Formal indices such as the mFI-5 were infrequently studied and did not consistently outperform KPS, mRS, ASA class, comorbidity burden, or baseline independence. The more clinically relevant construct may therefore be multidimensional geriatric reserve, incorporating function, comorbidity, cognition, nutritional status or sarcopenia, mobility, physiologic fitness, independence, and social support. Prospective validation of such a multidimensional assessment is needed before a standardized selection algorithm can be recommended [7,8,9,10].

### 4.2. Glioma: Surgery Within a Multimodal Treatment Pathway

The glioma literature contained the greatest number of adjusted survival analyses. GTR or source-defined maximal resection was frequently associated with longer overall survival in registry studies, whereas STR was less consistently associated with favorable outcomes, and institutional findings were more heterogeneous. These associations remain vulnerable to selection bias because registry datasets generally lacked performance status, frailty, detailed tumor anatomy, postoperative neurologic status, and reasons for treatment selection. Accordingly, greater resection should not be interpreted as causally improving survival. Its potential value appears conditional on achieving meaningful cytoreduction without substantial neurologic or functional decline [12,14,21,40,50].

Postoperative therapy was the most reproducible prognostic correlate across the glioma literature: every age-specific multivariable model evaluating radiotherapy, temozolomide, or combined therapy identified at least one adjuvant-treatment variable associated with longer survival. Postoperative complications, KPS decline, prolonged recovery, and non-home discharge may reduce the feasibility of treatment completion. Thus, maximal safe resection is clinically valuable only insofar as it preserves sufficient neurologic and functional status to permit progression through the multimodal treatment pathway [6,10,22,42,47].

Molecular classification also limits cross-era comparability. Many older glioma reports predated routine IDH and MGMT characterization and classified tumors primarily according to histopathology; consequently, historical “glioblastoma” and diffuse glioma cohorts may not map directly onto contemporary molecular entities. MGMT methylation appeared to modify treatment or resection associations in several more recent studies, although the evidence remained retrospective and limited. Future studies should therefore evaluate molecular subtype, achievable residual tumor volume, postoperative function, and adjuvant-treatment completion jointly rather than interpreting extent of resection in isolation.

### 4.3. Intracranial Meningioma: Function, Independence, and Competing Mortality

For intracranial meningioma, the principal clinical question differed from that in glioma. Many tumors were presumed benign or slow-growing, making symptom relief, preservation of independence, and avoidance of treatment-related disability at least as important as overall survival. Selected very elderly patients frequently improved or remained functionally stable after surgery, and several cohorts demonstrated improvement in neurologic status or mRS. However, contemporary multicenter studies also reported substantial rates of postoperative complications, new neurologic deficits, functional decline, rehabilitation dependence, and one-year mortality. Thus, the literature does not support characterizing meningioma surgery in the very elderly as uniformly safe or functionally restorative [7,15,65].

Baseline KPS, ASA class, cardiovascular or overall comorbidity burden, tumor size, edema, and the probability of a new postoperative neurologic deficit emerged as recurrent risk factors. The strongest associations often involved postoperative function rather than chronological age. In particular, a new neurologic deficit was associated with long-term dependency, emphasizing that the relevant operative objective is not simply removal of tumor but preservation of function. These findings support a symptom-directed, maximal-safe-resection strategy rather than anatomical completeness at any cost, especially for presumed benign disease [7,8,62,64].

The oncologic literature suggests that the value of gross-total resection may differ by tumor grade. Gross-total resection was not associated with longer overall survival in one mixed, predominantly low-grade population, whereas it was associated with more favorable survival in high-grade or atypical-meningioma cohorts. Postoperative radiotherapy was similarly associated with longer survival in selected atypical or mixed-histology analyses but not in all high-grade cohorts. These findings should be interpreted cautiously because the reports arose from repeatedly used database families, treated subgroups were small, and overall survival was the primary endpoint. In very elderly patients with benign or indolent meningioma, overall survival may be driven more strongly by competing non-tumor mortality than by local tumor control. The absence of age-specific progression, recurrence, meningioma-specific mortality, and treatment-burden outcomes limits the ability to determine whether more intensive treatment meaningfully improves the patient’s remaining life [20,63,69,70].

### 4.4. Sellar and Suprasellar Tumors: Visual Benefit Without a Requirement for Radical Resection

Among sellar tumors, visual improvement or preservation was the most consistently reported benefit of surgery. Across institutional cohorts, most patients with preoperative visual deficits improved or remained stable after transsphenoidal surgery. In contrast, recovery of pre-existing pituitary dysfunction was uncommon. New permanent endocrine morbidity, particularly persistent diabetes insipidus, appeared infrequent in the reported cohorts, although transient electrolyte and water-balance disturbances were observed [3,71,72,73,74].

The sellar literature provided one of the clearest examples of why the operative endpoint should be aligned with treatment intent. Durable visual decompression and tumor control were frequently achieved without gross-total resection. In several cohorts, complete removal was uncommon, yet visual outcomes and medium-term control remained favorable. These findings suggest that adequate optic-pathway decompression and preservation of pituitary and neurovascular structures may be more clinically relevant than radiographic completeness in selected very elderly patients [3,72].

At the same time, extended skull-base approaches were associated with greater complication risk, and the available reports reflected highly selected patients treated at experienced centers. The findings therefore cannot be generalized to all very elderly patients with giant, invasive, or anatomically complex tumors. The contrasting survival associations reported in registry analyses of pituitary adenoma and craniopharyngioma further illustrate the limitations of using overall survival to guide operative extent. Neither registry contained the visual, endocrine, hypothalamic, functional, or treatment-selection data required to explain these associations [23,24].

### 4.5. Spinal Tumors and Vestibular Schwannoma: Disease-Specific Functional Goals

Evidence regarding spinal and primary axial tumors was limited to three reports addressing biologically and anatomically distinct diseases. In spinal meningioma, surgery was associated with improvement in motor and ambulatory-disability scores, suggesting that progressive myelopathy may remain reversible even in octogenarians. However, systemic complications and early mortality were clinically important, and comorbidity burden predicted adverse events. For spinal ependymoma and spinal or axial osteosarcoma, surgery was associated with longer survival in registry analyses, but neurologic outcomes, operative morbidity, extent or quality of resection, and patient-selection variables were incomplete or absent. These survival associations should therefore be regarded as hypothesis-generating rather than causal evidence [4,9,76].

The vestibular schwannoma literature similarly reflected highly selected patients with large, progressive, compressive, or severely symptomatic tumors. Surgery was not primarily undertaken to preserve hearing, which was absent or severely impaired in most patients before surgery and was not preserved postoperatively. The more relevant potential benefits were relief of imbalance, trigeminal symptoms, hydrocephalus, or brainstem compression and eventual preservation or restoration of independence. Facial-nerve outcomes varied substantially between the two cohorts, indicating considerable uncertainty regarding the expected neurologic cost for an individual patient [5,16].

Both vestibular schwannoma cohorts frequently accepted planned residual tumors to reduce the risk of cranial-nerve injury. This approach may be reasonable when the goal is functional rescue rather than anatomical cure, but residual disease is not a completed treatment pathway. Progression, recurrence, and one late tumor-related death were reported, underscoring the need for long-term imaging and a predefined salvage strategy. The feasibility of surveillance and stereotactic radiosurgery should therefore be considered before planned subtotal resection [5,16].

### 4.6. A Tumor-Specific Definition of Benefit

Taken together, the findings demonstrate that “benefit from surgery” cannot be defined uniformly across primary central nervous system tumors in the very elderly. For glioblastoma, the principal goal may be survival extension while preserving the ability to complete chemoradiotherapy. For presumed benign meningioma, maintenance or restoration of independence may be more meaningful than overall survival. For pituitary tumors, visual decompression and durable control dominate. For spinal meningioma, ambulation and motor recovery are central. For vestibular schwannoma, the relevant benefit may be relief of mass effect or imbalance while preserving facial function.

This heterogeneity has implications for both clinical care and research. Gross-total resection, overall survival, and perioperative mortality cannot serve as universal measures of treatment success. A technically complete operation may be of limited value if it produces a deficit that prevents independent living or subsequent therapy. Conversely, subtotal resection may provide substantial benefit when it safely relieves compression and is paired with appropriate surveillance or adjuvant treatment. Operative success should therefore be judged relative to the specific harm the intervention is intended to prevent and the patient’s acceptable tradeoffs.

The evidence-informed framework developed from this review emphasizes six sequential considerations: defining tumor-specific urgency and intent; establishing physiologic and functional reserve; estimating achievable rather than theoretical surgical benefit; modeling the postoperative treatment and recovery pathway; comparing lower-burden alternatives; and aligning the recommendation with the patient’s remaining-life priorities (Table 5). This framework is intended to organize decision-making and should not be interpreted as a validated clinical prediction tool.

### 4.7. Health-System, Ethical, and Cultural Context of Surgical Decision-Making

The decision to offer neurosurgical intervention to a very elderly patient does not depend solely on tumor biology or operative risk. The included literature originated from diverse institutional and national settings, but few reports directly evaluated how health-system characteristics, access to subspecialty care, rehabilitation resources, availability of adjuvant therapy, or postacute-care infrastructure influenced treatment selection. These factors may be particularly important in older adults because the value of an operation may depend on timely rehabilitation, safe discharge, and access to subsequent radiotherapy, systemic therapy, surveillance, or salvage treatment. Consequently, treatment patterns observed in registry or tertiary-center studies may not be directly generalizable to health systems with different referral structures, resource availability, or thresholds for operative intervention.

Ethical considerations are similarly central. Decisions should balance potential survival or neurologic benefit against perioperative morbidity, loss of independence, treatment burden, and the patient’s remaining-life priorities. Chronological age alone should neither exclude a patient from potentially beneficial treatment nor justify an intervention whose expected burdens are unlikely to achieve a patient-valued goal. Assessment of decision-making capacity, elicitation of treatment preferences, involvement of surrogate decision-makers when appropriate, and explicit discussion of acceptable functional outcomes are therefore important components of shared decision-making.

Cultural differences may further influence how patients and families weigh longevity, independence, disability, treatment burden, and family involvement in decision-making. However, these domains were rarely measured in the reviewed literature, preventing meaningful comparison across cultural settings. Future prospective studies should therefore capture health-system characteristics, socioeconomic and access variables, patient and caregiver preferences, and culturally relevant measures of decision-making alongside conventional clinical outcomes.

From the authors’ experience within academic neurosurgical environments, these decisions are best approached as multidisciplinary and longitudinal rather than as isolated determinations of operative candidacy. Our practical strategy is to first define the tumor-specific purpose of intervention; assess baseline neurologic function, independence, comorbidity, frailty, cognition, and anticipated rehabilitation potential; estimate an achievable rather than purely anatomical surgical endpoint; and determine whether postoperative function is likely to permit the subsequent treatment pathway. Complex cases may additionally benefit from multidisciplinary input from neuro-oncology, radiation oncology, geriatrics, rehabilitation, palliative care, and other relevant specialties. When the anticipated benefit is uncertain, explicit discussion with patients and families regarding acceptable disability, treatment burden, alternatives to surgery, and goals for the patient’s remaining life is particularly important. This approach reflects the authors’ practice perspective and should not be interpreted as a formally validated institutional protocol.

### 4.8. Evidence Gaps and Future Research

The most important evidence gaps were not limited to small sample sizes. The literature rarely measured whether patients remained cognitively intact, returned home, recovered their prior independence, experienced acceptable quality of life, or imposed substantial caregiver burden. These omissions are particularly consequential in very elderly patients, for whom treatment burden and time spent in hospitals or rehabilitation facilities may be as important as survival duration.

Future studies should treat functional independence, cognition, patient-reported quality of life, days at home, caregiver burden, and goal concordance as core outcomes rather than ancillary endpoints. These measures should be assessed prospectively at baseline, discharge, 30 and 90 days, and longer-term follow-up alongside survival, complications, symptom relief, and treatment completion. Such outcomes may more directly characterize whether neurosurgical intervention improves the remaining life of very elderly patients rather than merely extending its duration.

Prospective multicenter registries should also collect direct measures of geriatric reserve, including frailty phenotype, cognition, gait, nutrition, sarcopenia, social support, and baseline living situation. Comparative-effectiveness studies are needed to evaluate surgery against observation, biopsy, stereotactic radiosurgery, conventional radiotherapy, and medical management. Because randomized trials may be difficult in many of these settings, carefully designed prospective cohorts, target-trial emulations, hierarchical models, and center-clustered external validation may provide more credible estimates than additional single-database retrospective analyses.

The repeated use of SEER, NCDB, and other national databases also requires greater attention. Multiple reports likely included overlapping patients, and concordant associations should not be interpreted as independent replication. Future reviews and database studies should explicitly report overlap, study periods, inclusion criteria, and whether analyses represent companion or nested cohorts. Because these reports were observational and several registry analyses reused overlapping source populations, concordant survival associations were interpreted neither as causal treatment effects nor as independent patient-level replication.

### 4.9. Limitations

This review has several limitations. First, as a scoping review, it was designed to map and synthesize the literature rather than estimate a pooled treatment effect. The substantial clinical and methodological heterogeneity across tumors, interventions, age thresholds, comparators, and outcome definitions precluded meaningful meta-analysis. The absence of pooling should not be interpreted as evidence that treatment effects do not exist, but rather that the available reports could not support a single quantitative estimate.

Second, nearly all included reports were retrospective and were therefore vulnerable to selection bias, confounding by indication, immortal-time bias, incomplete adjustment, and missing data. Patients selected for surgery were likely healthier, more independent, or more likely to have resectable tumors and receive subsequent treatment than patients managed nonoperatively. Even multivariable adjustment could not fully address unmeasured differences in functional reserve, anatomy, patient preference, or clinician judgment. Accordingly, associations between surgery, extent of resection, radiotherapy, and survival should not be interpreted causally.

Third, outcome definitions and follow-up intervals varied substantially. Complications were inconsistently defined, and functional outcomes ranged from KPS and mRS to discharge destination, motor scores, visual status, and cranial-nerve function. This heterogeneity limited direct comparison and required tumor-specific narrative synthesis.

Fourth, national database reports frequently arose from overlapping source families. Although confirmed and likely overlap clusters were identified, patient-level overlap could not always be established. Report counts therefore overstate the number of fully independent patient populations represented in the literature.

Fifth, the evidence base was disproportionately concentrated in glioma and meningioma. Conclusions regarding sellar, spinal, vestibular schwannoma, and other rare tumors were based on few reports and highly selected patients. The findings in these categories should be viewed as descriptive and hypothesis-generating.

Sixth, five reports could not be retrieved, and reports available only as abstracts were excluded when eligibility and outcome data could not be confirmed. Relevant unpublished or non-English evidence may also have been missed despite the broad database search.

Finally, the review included reports using different definitions of “very elderly,” including thresholds of 75, 80, and 85 years. Although only separately extractable outcomes for patients aged 75 years or older were retained, residual heterogeneity in age composition may affect comparability across reports.

## 5. Conclusions

Neurosurgical intervention may be appropriate for selected adults aged 75 years or older with primary central nervous system tumors, but meaningful benefit is tumor-specific and cannot be inferred from chronological age or extent of resection alone. Decisions should integrate tumor biology and urgency, functional and physiologic reserve, achievable surgical goals, preservation of neurologic function, feasibility of subsequent therapy, and patient priorities. Prospective multicenter studies incorporating geriatric assessment and patient-centered outcomes are needed before these principles can support validated selection tools or causal treatment recommendations.

## Figures and Tables

**Table 1:** Evidence architecture and characteristics of the included reports (*N* = 80).

Characteristic	Reports, *n*/*N*	%
**Tumor category**		
Glioma/glioblastoma	37/80	46.3
Intracranial meningioma	25/80	31.3
Sellar/suprasellar tumors	8/80	10.0
Mixed/other primary intracranial tumors	5/80	6.3
Spinal/primary vertebral tumors	3/80	3.8
Vestibular schwannoma	2/80	2.5
**Publication period**		
1995–2009	7/80	8.8
2010–2014	3/80	3.8
2015–2019	19/80	23.8
2020–2024	38/80	47.5
2025–July 2026	13/80	16.3
**Study design**		
Retrospective clinical cohort or case series	46/80	57.5
Retrospective database or registry analysis	29/80	36.3
Mixed prospective–retrospective cohort	2/80	2.5
Retrospective analysis of a prospectively maintained database	1/80	1.3
Prospective observational cohort	1/80	1.3
Prospective single-arm phase II study	1/80	1.3
**Primary data-source architecture**		
Population cancer registry	18/80	22.5
National clinical or administrative database	11/80	13.8
Multicenter clinical cohort	7/80	8.8
Single-center clinical cohort	24/80	30.0
Institutional clinical cohort; number of centers not stated	20/80	25.0
**Selected evidence-architecture metrics**		
Glioma or meningioma reports	62/80	77.5
Reports published in 2015 or later	70/80	87.5
Purely retrospective reports	75/80	93.8
Prospectively conducted reports	2/80	2.5
Randomized treatment-comparison studies	0/80	0.0
Explicitly multicenter reports among clinical cohorts	7/51	13.7
Database reports from repeatedly used source families	27/29	93.1

Note. Values are report-level counts and percentages; percentages may not total 100.0 because of rounding. The denominator is 80 unless a different denominator is shown. Clinical cohorts comprised 51 reports, of which 7 (13.7%) were explicitly multicenter. National or population databases comprised 29 reports, of which 27 (93.1%) came from repeatedly used source families: SEER/SEER-Medicare, the National Cancer Database, ACS-NSQIP, or the Japanese Diagnosis Procedure Combination database. Counts represent publications rather than confirmed independent patient cohorts; overlapping reports were retained for distinct research questions but were not treated as independent replication.

**Table 2:** Age-specific evidence on surgical strategy and the treatment pathway in very elderly patients with glioma.

**Panel A. Age-specific comparative evidence (13 evidence units from 12 reports)**					
**Study/Cohort**	**Age-Specific Population and Source**	**Surgical Comparison**	**Survival Finding**	**Functional or Treatment-Pathway Finding**	**Principal Limitation**
Karsy et al., 2018 [6]	GBM; age ≥ 75; *n* = 82; single-center retrospective cohort	No surgery vs. biopsy vs. STR vs. GTR	Mean OS: 0.8, 3.7, 5.0, and 12.1 months. EOR was not independently associated with OS (HR, 0.59; 95% CI, 0.29–1.20; *p* = 0.14).	Complications were associated with mortality (HR, 5.43; 95% CI, 1.73–17.04; *p* = 0.004), postoperative KPS decline, and loss of the EOR–survival gradient.	Small selected groups; no treatment-completion data; confounding by indication.
Ahmadipour et al., 2020 [12]	GBM; age 75–84; *n* = 85; single-center cohort; all received Stupp therapy	GTR or STR vs. stereotactic biopsy	GTR was associated with +3.900 months of adjusted survival vs. biopsy (95% CI, 1.472–6.329; *p* = 0.002); STR was not (+1.685 months; 95% CI, −2.660 to 6.029; *p* = 0.433).	Both GTR and STR were associated with greater postoperative KPS decline than biopsy.	Nonrandom selection; survival-time coefficient rather than HR; internal *p*-value discrepancy resolved using the table value.
Osawa et al., 2021 [37]	GBM; age ≥ 75; *n* = 31; single-center retrospective cohort	Resection (*n* = 20) vs. biopsy (*n* = 11)	Median OS: 11.5 vs. 6.77 months (*p* = 0.241); median PFS: 7.57 vs. 5.23 months (*p* = 0.131).	Postoperative neurologic deterioration occurred in 15% after resection and 0% after biopsy.	Very small cohort; no adjusted direct treatment estimate.
Lopez-Rivera et al., 2021 [18]	GBM; SEER 2000–2016; total *n* = 17,820; age-specific analyses for ≥75 and ≥80	Biopsy/local excision vs. STR vs. GTR vs. supratotal resection	Age ≥ 75: GTR HR, 0.61 and supratotal HR, 0.63. Age ≥ 80: STR HR, 0.83; GTR HR, 0.61; supratotal HR, 0.64; all *p* < 0.0001.	Functional, frailty, neurologic, and perioperative outcomes were not available.	Registry EOR coding; no performance or molecular data; substantial overlap with other SEER GBM analyses.
Li et al., 2022—institutional cohort [13]	GBM; age ≥ 75; *n* = 50; single-center retrospective cohort	Partial vs. subtotal vs. gross-total resection	Median OS: 83, 200, and 278 days. Versus partial resection: STR HR, 0.134 (95% CI, 0.022–0.831; *p* = 0.031); GTR HR, 0.100 (95% CI, 0.015–0.671; *p* = 0.018).	Complications occurred in 6%; EOR was not associated with new neurologic deficit.	Only four partial-resection patients; wide CIs and residual selection bias.
Li et al., 2022—SEER cohort [13]	GBM; age ≥ 75; *n* = 6079; SEER 2005–2016	No surgery vs. biopsy vs. subtotal/partial resection vs. GTR	Median OS: 2, 3, 4, and 6 months. Versus biopsy: subtotal/partial HR, 0.796 (95% CI, 0.665–0.951; *p* = 0.012); GTR HR, 0.566 (95% CI, 0.472–0.680; *p* < 0.001).	Three-month mortality: 58.4%, 43.1%, 36.5%, and 28.2%, respectively.	No performance, frailty, molecular, complication, or treatment-completion variables; SEER overlap.
Niare et al., 2022 [40]	GBM; age ≥ 80; *n* = 117; single-center retrospective cohort	Resection (*n* = 57) vs. biopsy (*n* = 60)	Median OS: 9.5 vs. 4.0 months (*p* < 0.001); resection plus Stupp therapy: 17.5 months.	Overall complication rates were similar; mean KPS remained stable in the selected resection group.	Profound baseline imbalance; no adjusted direct resection-versus-biopsy estimate.
Zhao et al., 2022 [41]	GBM; age ≥ 80; *n* = 3309; SEER 2005–2016 with competing-risk and propensity analyses	No surgery, biopsy/partial, STR, and GTR; radical vs. nonradical surgery	Median OS: 2, 3, 3, and 5 months. Propensity-matched radical surgery was associated with lower mortality (subdistribution HR, 0.729; *p* < 0.001).	One-year OS was 11% after radical vs. 6% after nonradical surgery; the advantage was attenuated by conditional year 3.	Overlapping SEER population and residual confounding despite matching.
Horowitz et al., 2024 [21]	GBM; age ≥ 80; *n* = 1868; SEER 2000–2020	Biopsy vs. STR vs. GTR	Versus biopsy: STR HR, 0.997 (95% CI, 0.890–1.117; *p* = 0.957); GTR HR, 0.696 (95% CI, 0.619–0.781; *p* < 0.001).	No functional, perioperative, cognitive, or quality-of-life outcomes were available.	No performance, comorbidity, MGMT, complication, or treatment-intent data; SEER overlap.
Singh et al., 2026 [49]	Intracranial low-grade glioma; age ≥ 85; *n* = 182; SEER 2000–2022	No resection vs. STR vs. GTR	Median OS: 2.0, 4.5, and 5.5 months. Versus no resection: STR HR, 0.628 (95% CI, 0.416–0.949; *p* = 0.027); GTR HR, 0.543 (95% CI, 0.311–0.950; *p* = 0.033).	No functional, cognitive, perioperative, or quality-of-life outcomes were available.	Likely histologic/molecular misclassification; only 52 resected patients; no performance data.
Jankovic et al., 2026 [50]	IDH-wildtype GBM; age ≥ 75; *n* = 108; single-center retrospective cohort	Biopsy vs. submaximal vs. maximal resection	With submaximal resection as reference: maximal resection HR, 0.379 (95% CI, 0.218–0.659; *p* < 0.001); biopsy HR, 0.768 (95% CI, 0.424–1.390; *p* = 0.383).	Maximal resection was not associated with greater postoperative deterioration; benefit was strongest in MGMT-methylated tumors.	Anatomic and performance differences between treatment groups; supports maximal vs. submaximal resection, not a causal biopsy comparison.
Ono et al., 2026 [51]	GBM; age ≥ 80; *n* = 31; single-center frail cohort	Resection vs. biopsy vs. treatment based on clinical diagnosis	Median OS: 771, 389, and 631 days (*p* = 0.25). Resection HR, 0.50 (95% CI, 0.11–2.18; *p* = 0.36); biopsy HR, 2.17 (95% CI, 0.85–5.54; *p* = 0.11).	Surgical intervention was associated with lower odds of KPS improvement (OR, 0.12; *p* = 0.020); biopsy was associated with lower odds of home discharge (OR, 0.12; *p* = 0.012).	Tiny nonrandom groups; clinical-diagnosis group received bevacizumab; severe confounding.
Song et al., 2026—octogenarian subgroup [14]	Lobar GBM; age ≥ 80; *n* = 27 within a single-surgeon cohort	Resection vs. biopsy	Median OS: 6.5 vs. 3.0 months (*p* = 0.035). Adjusted HR for resection, 0.28 (95% CI, 0.10–0.81; *p* = 0.018).	Among surviving resection patients, 75.0%, 80.0%, and 71.4% had ECOG 0–1 at 3, 6, and 12 months.	Small lobar-tumor subgroup; functional results conditioned on survival; EOR effect attenuated in good-function subgroup.
**Panel B. Cross-study selection and treatment-pathway findings**					
**Domain**	**Evidence Summary**			**Numerator/Denominator**	**Interpretation**
Age-specific surgical evidence	Twelve reports provided 13 evidence units because one report contained separate institutional and SEER cohorts.			12/37 reports; 13 units	Comparative evidence represented one-third of the glioma corpus.
Adjusted surgical association by source	Adjusted survival associations were universal in registry units but present in half of institutional units.			Registry: 5/5; institutional: 4/8	Registry consistency may reflect scale plus residual selection and coding bias.
Functional or perioperative reporting	Seven evidence units reported functional or perioperative outcomes.			7/13 units	Neurologic and functional tradeoffs were less commonly measured than survival.
Chronological age	Age was independently associated with survival in one of six relevant multivariable models.			1/6 models	Age alone was less informative than reserve, anatomy, and treatment feasibility.
Baseline performance status	KPS or ECOG was independently associated with survival in four of eight models.			4/8 models	Performance was important but insufficient as a stand-alone selection rule.
Adjuvant treatment	Every model, including postoperative RT, TMZ, or combined therapy, identified at least one treatment variable associated with longer survival.			9/9 models	Surgical value cannot be separated from the ability to complete postoperative therapy.
Postoperative pathway harm	Five studies linked complications, KPS decline, non-home discharge, or prolonged recovery to an adverse pathway.			5/20 reports	Recovery may mediate or negate the oncologic value of cytoreduction.
MGMT modification	Three studies reported greater treatment or resection benefit in MGMT-methylated tumors.			3/20 reports	Molecular biology may concentrate treatment value.

Note. Associations are observational and should not be interpreted as causal treatment effects. The primary synthesis was restricted to findings directly applicable to a cohort aged ≥ 75 years or a separately extractable age-eligible subgroup. One report contributed separate institutional and SEER evidence units. Several SEER analyses likely reused overlapping patients and were retained for distinct questions but were not treated as independent replication. HR indicates hazard ratio; CI, confidence interval; ECOG, Eastern Cooperative Oncology Group performance status; EOR, extent of resection; GBM, glioblastoma; GTR, gross-total resection; IDH, isocitrate dehydrogenase; KPS, Karnofsky Performance Status; MGMT, O6-methylguanine-DNA methyltransferase; OS, overall survival; PFS, progression-free survival; SEER, Surveillance, Epidemiology, and End Results; STR, subtotal resection; TMZ, temozolomide.

**Table 3:** Surgical safety, functional outcomes, and histology-specific oncologic management in very elderly patients with intracranial meningioma.

**Panel A. Age-specific surgical safety, functional outcomes, and patient-selection evidence (18 reports; 17 source populations)**					
**Study/Cohort**	**Age-Specific Population and Source**	**Functional or Independence Finding**	**Safety or Mortality Finding**	**Key Quantitative Predictor Finding(s)**	**Principal Limitation**
Mastronardi et al., 1995 [53]	Intracranial meningioma; age ≥ 80; *n* = 17; single-center historical series	Preoperative functional limitation was central to risk stratification.	ASA III was associated with major morbidity (*p* = 0.020) and mortality (*p* = 0.005).	KPS < 70 was associated with adverse outcome (*p* = 0.010); diameter > 5 cm increased morbidity (*p* = 0.031).	Very small historical cohort; unadjusted associations.
Sacko et al., 2007 [54]	Ninth decade; *n* = 74; single-center retrospective cohort	KPS improved during the first postoperative year in 39 patients (53%).	No perioperative mortality; complications 9.4%; 1-year mortality 9.4%.	Better outcomes with KPS ≥ 60, ASA I–II, noncritical location, and absent/moderate edema; large tumors, critical location, severe edema, and total excision were associated with complications.	Historical cohort; predictor analysis largely unadjusted.
Riffaud et al., 2007 [55]	Age > 80; *n* = 11; single-center retrospective series	At 3 months, 6 improved, 4 were unchanged, and 1 worsened. At 1 year, 8/10 survivors had KPS > 80 and were self-sufficient.	No perioperative mortality; one perioperative hemiplegia; one death by 1 year.	No formal multivariable model.	Only 11 highly selected patients.
Yamamoto et al., 2017 [57]	Age ≥ 75 subgroup; *n* = 16; single-center comparison with younger patients	Rehabilitation/convalescent placement: 31.2% vs. 9.3% in younger patients (*p* = 0.042).	No postoperative mortality; one cranial-nerve palsy.	Simpson grade III–V predicted complications: OR, 5.662 (95% CI, 1.323–24.236; *p* = 0.0194). Age ≥ 75, skull-base site, size, KPS, and ASA were not independently associated.	Small age-eligible subgroup; wide CI and confounding by operative complexity.
Steinberger et al., 2018 [58]	Supratentorial meningioma; ACS-NSQIP *n* = 1568; age > 80 subgroup	Octogenarians had greater baseline dependence; longitudinal recovery was unavailable.	Age > 80 predicted any complication (OR, 2.374; 95% CI, 1.3–4.4), 30-day death (OR, 15.7; 95% CI, 3.0–81.0), and LOS > 5 days (OR, 3.2; 95% CI, 1.8–5.6). Observed mortality was 8.6%.	Age effect was estimated relative to patients aged 18–50.	Very young reference group; no anatomy, EOR, or long-term function.
Dobran et al., 2018 [59]	Age > 80; *n* = 25; single-center retrospective cohort	Median KPS increased from 74.3 preoperatively to 82 at 6 months.	One-month mortality was 8% (2/25). Duration > 240 min correlated with mortality (*p* = 0.0421); mortality was lower with ASA II than ASA III (*p* = 0.038).	Operative duration (*p* = 0.006) and lesion size > 4 cm (*p* = 0.02) were associated with clinical improvement.	Small cohort; no stable multivariable model.
Corniola et al., 2020 [61]	Age ≥ 80 *n* = 49 vs. age 70–79 *n* = 272; mixed retrospective/prospective institutional cohort	Preoperative KPS ≥ 70 was associated with longer survival.	Complications were similar; 30-day mortality 8.2% vs. 4.1% (*p* = 0.25).	Female sex (OR, 0.36; 95% CI, 0.14–0.93; *p* = 0.03) and KPS ≥ 70 (OR, 0.27; 95% CI, 0.10–0.72; *p* = 0.009) were associated with survival.	Operated patients only; strong selection and competing mortality.
Hadanny et al., 2020 [62]	Age ≥ 80 *n* = 31; matched younger *n* = 31; single-center cohort	Matching incorporated KPS, tumor size, and location.	30-day mortality 6.5% vs. 0%; 6-month mortality 12.9% vs. 3.2%; complications 77.4% vs. 74.2%.	ASA class was the sole predictor of any complication: OR, 2.219 (95% CI, 1.024–4.811; *p* = 0.04).	Small matched cohort; broad complication definition.
Ekaireb et al., 2021 [64]	Age ≥ 75; *n* = 103; single-center retrospective cohort	No standardized longitudinal independence outcome.	Any complication 31.1%; major complication 15.5%; increasing age did not predict complications.	Cardiovascular comorbidity predicted any complication: OR, 3.94 (95% CI, 1.05–14.76; *p* = 0.0238). Male sex predicted major complications: OR, 3.78 (95% CI, 1.20–11.93; *p* = 0.0212).	No untreated comparator or standardized postoperative function.
Joubert et al., 2021 [65]	Age > 80; *n* = 37; single-center retrospective cohort	At discharge, KPS improved in 21, was stable in 10, and worsened in 6; mean gain among improved patients was 18.1 points.	One-year mortality 2.7%; tumor control in 33 patients.	Improved/stable KPS was associated with shorter LOS, Clavien–Dindo grade < 2, and lower preoperative KPS.	Selected operated cohort; limited adjusted analysis.
Ahmeti et al., 2021 [66]	Age ≥ 80 subgroup within *n* = 768; single-center age-stratified cohort	Immediate KPS did not improve significantly in patients ≥ 80 (*p* = 0.753); longer-term improvement was reported for the full cohort.	Morbidity increased with age; bleeding, palsy, speech disorder, pneumonia, and renal insufficiency were age-associated.	The age ≥ 80 subgroup had lower preoperative KPS and greater comorbidity.	Many results pooled patients aged ≥ 65; limited age-specific inference.
Ikawa et al., 2022 [11]	Japanese DPC surgical cohort *n* = 8138; age ≥ 75 subgroup	mFI-5 did not predict Barthel Index deterioration in patients aged ≥ 75.	mFI-5 ≥ 2 did not predict any complication in the age ≥ 75 subgroup.	Chronological age, rather than mFI-5, remained associated with worsening outcomes.	Administrative frailty coding; identical cohort to Ikawa et al., 2024 [19].
Kusyk et al., 2022 [67]	Skull-base meningioma; octogenarians; *n* = 14; case series	Approximately half were discharged home or to rehabilitation; one required tracheostomy.	30-day mortality was 14%.	No measured characteristic significantly predicted poor outcome.	Only 14 patients; very limited statistical power.
Maiuri et al., 2023 [68]	Age ≥ 80 *n* = 17 vs. younger groups; single-center cohort	No standardized longitudinal independence outcome.	Neurologic deficits and acute bronchopneumonia were more frequent in patients ≥ 80; hematoma, pulmonary embolism, cardiac ischemia, and death were not significantly different.	The age ≥ 80 group had more comorbidities and adverse pathological markers.	Only 17 age-eligible patients; multiple comparisons and limited adjustment.
Filippidis et al., 2023 [15]	Age 80–89; *n* = 30; single-center cohort	One-year mRS improved significantly relative to baseline (McNemar chi-square, 9.6; *p* = 0.001946).	One-year survival 86.3%; complications 16.7%; transient new deficits 30%.	Age, mRS, mFI-5, grade, size, EOR, and admission KPS did not significantly affect survival.	Small cohort; survivor and follow-up selection.
Schwartz et al., 2024 [7]	Supratentorial meningioma; age ≥ 80; *n* = 262; international multicenter cohort	At 1 year, 38.5% improved, 33.2% were unchanged, and 28.2% were worse, including deaths. Among patients with poor baseline status and large edema, 58.3% benefited.	Complications 42.4%; 90-day mortality 9.0%; 1-year mortality 13.2%.	Each year of age increased 90-day and 1-year mortality risk by 44% (95% CI, 23–70%). Diameter ≥ 5 cm predicted complications (OR, 1.87; 95% CI, 1.12–3.13); large volume OR, 2.35 (95% CI, 1.01–5.50).	Retrospective multicenter heterogeneity; supratentorial tumors only.
Ikawa et al., 2024 [19]	Japanese DPC surgical cohort *n* = 8138; age ≥ 75 subgroup	Advanced age predicted Barthel Index deterioration: OR, 3.26 (95% CI, 2.69–3.95).	Advanced age was not independently associated with in-hospital mortality.	Admission BI 60–80 increased deterioration risk; BI < 60 showed an inverse association (OR, 0.47; 95% CI, 0.32–0.69), likely reflecting floor effects and selection.	Identical DPC cohort to Ikawa et al., 2022 [11]; administrative outcomes.
Delgado-Lopez et al., 2025 [8]	Age ≥ 80; *n* = 189; multicenter retrospective cohort	Mean KPS declined from 73.5 to 63.5 at 12 months (*p* < 0.001); 27.1% remained independent.	New deficits 23.8%; complications 42.8%; 30-day mortality 4.2%; 1-year mortality 15.8%.	Preoperative KPS < 70 predicted unfavorable outcome (OR, 3.10; 95% CI, 1.44–6.68; *p* = 0.004). KPS < 70 (OR, 14.45; 95% CI, 5.64–37.03) and new deficits (OR, 4.79; 95% CI, 1.59–14.38) predicted 12-month KPS < 70.	Retrospective multicenter heterogeneity; composite outcome.
**Panel B. Histology-specific oncologic management and population-level survival evidence (6 reports)**					
**Study/Histology**	**Age-Specific Population and Evidence Role**	**Treatment Utilization/Comparison**	**Surgery or EOR Finding**	**Radiotherapy and Survival Finding**	**Principal Limitation**
Cahill et al., 2011 Nonmalignant [56]	SEER 2004–2007; age ≥ 80 category *n* = 2132; contextual because treatment effect was pooled across ages	Among 2098 with known treatment, 245 underwent resection (11.7%); patients ≥ 80 were least likely to undergo surgery.	Pooled histologically confirmed cohort: resection vs. biopsy/no surgery HR, 0.75 (95% CI, 0.59–0.95).	No direct age-specific RT estimate. Three-year survival across all ages was 93.4% after resection vs. 88.3% after biopsy/no resection.	Only 55% histologically confirmed; no data on function, comorbidity, recurrence, or age-specific treatment effect.
Achey et al., 2019 Nonmalignant and malignant [2]	CBTRUS incidence/SEER survival 2005–2015; age 65+; contextual treatment effects pooled across ages	No direct octogenarian treatment-effect model.	Nonmalignant pooled STR vs. GTR HR, 1.123 (95% CI, 0.986–1.280; *p* = 0.082).	Nonmalignant median OS: 92 months at 75–79, 63 months at 80–84, and 32 months at ≥ 85. GTR + RT vs. GTR HR, 1.569 (95% CI, 1.156–2.130; *p* = 0.004); STR + RT vs. GTR HR, 1.434 (95% CI, 1.062–1.934; *p* = 0.019). Malignant RT vs. none HR, 1.019 (*p* = 0.890).	OS rather than local control or cause-specific survival; treatment selection strongly confounded.
Liu et al., 2021 High-grade [63]	SEER 1990–2016; age ≥ 80; *n* = 678; direct octogenarian treatment estimates	Observation 34.1%; RT alone 4.1%; surgery+/−RT 61.8%. Among surgical patients, GTR 49.4% and STR 50.6%.	Surgery+/−RT vs. observation HR, 0.807 (95% CI, 0.666–0.977; *p* = 0.028). STR vs. GTR HR, 1.275 (*p* = 0.043).	Median OS: surgery 32 months, observation 20 months, RT alone 11 months. RT alone vs. observation HR, 1.066 (95% CI, 0.688–1.651; *p* = 0.775); postoperative RT was nonsignificant.	SEER-era/coding heterogeneity; no performance, complications, recurrence, local control, or disease-specific death.
Karabacak et al., 2024 WHO grade II–III [69]	NCDB 2010–2017; octogenarians *n* = 911; contextual because treatment HRs were pooled across ages 60–89	No surgery 20.6%; among known EOR, GTR 66.6% and STR 33.4%; RT 19.2%. Odds of surgery (OR, 0.821) and RT (OR, 0.429) vs. patients aged 60–69.	Pooled GTR vs. no surgery HR, 0.80 (95% CI, 0.73–0.89; *p* < 0.001); STR vs. no surgery HR, 1.04 (*p* = 0.489).	Pooled RT HR, 0.79 (95% CI, 0.72–0.87; *p* < 0.001). Octogenarian age HR, 2.65 (95% CI, 2.36–2.97); LOS > 7 days OR, 1.326 (*p* = 0.009).	Mixed grade II–III cohort; no age-specific treatment effect on PFS, recurrence, function, or cause-specific survival.
Reddy et al., 2025 Predominantly grade I [20]	SEER 2000–2020; age ≥ 80; *n* = 590; direct very elderly estimates	No operation 22.9%; STR 29.7%; GTR 47.5%; RT 5.6% (33 patients).	GTR vs. STR HR, 1.003 (95% CI, 0.682–1.475; *p* = 0.987); Kaplan–Meier *p* = 0.84.	Median OS 20 months. RT HR, 0.212 (95% CI, 0.052–0.860; *p* = 0.030), but the treated group was very small and selected.	Only 33 RT recipients and 257 complete cases; no function, frailty, location, recurrence, local control, or cause-specific death.
Tang et al., 2025 WHO grade II atypical [70]	NCDB 2004–2019; surgically treated age >80; *n* = 382; direct postoperative estimates	GTR 58.9%; STR 41.1%; RT 14.13% vs. 26.5% at age 65–80.	STR vs. GTR HR, 1.34 (95% CI, 1.01–1.77; *p* = 0.04).	Adjuvant RT HR, 0.51 (95% CI, 0.33–0.80; *p* < 0.01); RT was not significant at age 65–80 (HR, 0.85; *p* = 0.11).	Surgical cohort only; NCDB cannot distinguish adjuvant from salvage RT or measure recurrence, PFS, performance, or cause-specific survival.
**Panel C. Cross-study synthesis**					
**Domain**		**Evidence Summary**		**Numerator/Denominator**	**Interpretation**
Primary function/safety evidence		Eighteen age-specific surgical reports represented 17 source populations because the 2022 and 2024 Japanese DPC reports analyzed the same 8138-patient cohort.		18 reports/17 populations	Publication count slightly overstates independent evidence.
Functional or discharge outcomes		Fourteen reports measured function, independence, or disposition, but instruments and follow-up periods varied substantially.		14/18 reports	Longitudinal independence was more commonly reported than in glioma but remained heterogeneous.
Long-term function		Only six reports assessed function at approximately 1 year.		6/18 reports	The most patient-relevant outcome was available in one-third of reports.
Patient-selection factors		Ten reports used adjusted analyses; six identified KPS, ASA/comorbidity, or admission function as independent predictors, and six identified tumor burden or anatomy as significant risk factors.		10/18 adjusted; 6 reserve; 6 tumor burden	Physiologic reserve and anatomy were generally more actionable than age alone.
Formal frailty measurement		Only three reports used mFI-type measures, and frailty indices did not consistently outperform KPS, ASA, comorbidity, or direct functional status.		3/18 reports	The evidence does not validate a frailty threshold for surgery.
Functional benefit and harm		Selected cohorts often improved or remained stable, but the largest multicenter supratentorial cohort found 28.2% worse at 1 year, and the 2025 multicenter cohort reported mean KPS decline with only 27.1% independent at 12 months.		2 largest contemporary cohorts	Surgical feasibility does not guarantee preservation of independence.
Low-grade oncologic management		In a predominantly grade I very elderly cohort, GTR did not improve OS over STR (HR, 1.003; *p* = 0.987).		1 direct report	Anatomical completeness should not be assumed to improve survival in presumed benign disease.
High-grade or atypical management		STR was associated with higher mortality than GTR in high-grade and atypical cohorts (HR, 1.275 and 1.34). RT was associated with longer OS in the atypical cohort but not in the earlier high-grade SEER cohort.		2 direct EOR reports; 3 direct RT reports	More intensive oncologic management may be relevant in aggressive histology, but estimates are observational and source-overlapping.
Oncologic endpoint limitations		All six population-level reports relied principally on OS; none provided age-specific progression/local-control or cause-specific treatment-effect models.		0/6 reports	Competing mortality and confounding by indication remain unresolved.

Note. Associations are observational and should not be interpreted as causal treatment effects. Panel A includes 18 age-specific surgical reports representing 17 source populations because Ikawa et al. (2022) [11] and Ikawa et al. (2024) [19] analyzed the identical Japanese Diagnosis Procedure Combination cohort. Panel B distinguishes direct age-specific treatment estimates from contextual reports in which treatment effects were pooled across broader age groups. Several SEER/CBTRUS and NCDB reports likely reused overlapping patients and were retained for distinct clinical questions but were not treated as independent replication. ASA indicates American Society of Anesthesiologists physical status; BI, Barthel Index; CBTRUS, Central Brain Tumor Registry of the United States; CI, confidence interval; DPC, Diagnosis Procedure Combination; EOR, extent of resection; GTR, gross-total resection; HR, hazard ratio; KPS, Karnofsky Performance Status; LOS, length of stay; mFI-5, 5-factor modified frailty index; mRS, modified Rankin Scale; NCDB, National Cancer Database; OR, odds ratio; OS, overall survival; RT, radiotherapy; SEER, Surveillance, Epidemiology, and End Results; STR, subtotal resection.

**Table 4:** Sellar/suprasellar, spinal, and vestibular schwannoma evidence in very elderly patients.

**Panel A. Sellar and suprasellar tumors: visual, endocrine, safety, and tumor-control evidence (8 reports)**					
**Study/Disease**	**Age-Specific Population and Source**	**Treatment Strategy/Indication**	**Functional or Tumor-Specific Outcome**	**Safety, Tumor Control, and Adjusted Findings**	**Principal Interpretation/Limitation**
Yunoue et al., 2014 Pituitary adenoma [71]	Age ≥ 80; *n* = 10; 9/10 nonfunctioning; ASA II–III; two-center retrospective series	Transsphenoidal surgery; total 7/10, subtotal 2/10, partial 1/10.	All 8 patients with preoperative visual-field deficits improved. Pre-existing anterior pituitary deficits showed no meaningful recovery.	No permanent morbidity or postoperative KPS deterioration; mean stay 11.5+/−3.6 days. No regrowth over mean of 37.9 months; one residual tumor received Gamma Knife.	Visual decompression was reliable, but endocrine recovery was poor. Very small, selected historical cohort.
Fujimoto et al., 2017 Nonfunctioning pituitary adenoma [72]	Age > 80; *n* = 12; single-center endoscopic series	Neurologic/visual mass-effect symptoms; total removal 2/12 (16.6%), reflecting a decompression-first strategy.	Vision improved in 11/12 (91.7%) and deteriorated in 1/12. New anterior hormonal deficiency occurred in 2/12; transient DI in 2; no persistent DI.	CSF leak 3/12 (25.0%); other complications 4/12 (33.3%); mean stay 19.0+/−8.5 days. Versus patients < 80: lower total removal (*p* = 0.016), higher CSF leak (*p* = 0.014), and higher other complications (*p* = 0.001), but similar visual improvement (*p* = 0.59). One residual regrew and required reoperation.	Visual benefit persisted despite limited resection, but anatomically complex tumors produced substantial recovery burden.
Chinezu et al., 2017 Nonfunctioning pituitary adenoma [73]	Age 80–85; *n* = 15; comparator age 65–75, *n* = 49; single-surgeon cohort	Endoscopic surgery; GTR 8/15 and STR 7/15.	Vision improved in 12/15, was stable in 2, and worsened in 1; improvement was more frequent than in the comparator group (*p* = 0.0012). One of 10 with normal preoperative function developed a new endocrine deficit; one of 4 partial deficits improved, and none normalized.	No deaths or CSF leaks; transient DI 1/15; electrolyte imbalance 4/15. No reoperation or radiosurgery during mean follow-up of 16.86 months; no age-group difference in complication distribution (*p* = 0.58) or stay (*p* = 0.91).	Age >80 alone did not preclude favorable visual outcomes in a highly selected expert-surgeon cohort.
Memel et al., 2019 Pituitary adenoma [74]	Age ≥ 80; *n* = 21; comparator age 70–79, *n* = 94; single-center transsphenoidal cohort	Transsphenoidal surgery for predominantly nonfunctioning, mass-effect tumors.	Vision worsened in 3/16 assessed octogenarians (18.8%) versus 2/67 comparators (3.0%; *p* = 0.0171). New panhypopituitarism occurred in 18.2% versus 7.4% (*p* = 0.1275); hyponatremia occurred in 2/19; no permanent DI.	Surgical complications 26.3% versus 7.9% (*p* = 0.0208); cranial-nerve palsy 10.53% versus 0% (*p* = 0.0296); no recorded complication in 52.63% versus 77.53% (*p* = 0.0432). Recurrence/progression 15.8% over mean follow-up of 27 months.	Modern short-stay surgery remained feasible, but octogenarians had a greater surgical and visual complication burden; denominators varied.
Teramoto et al., 2023 Nonfunctioning pituitary adenoma [75]	Age ≥ 75; *n* = 29; ASA I–II only; endocrine-testing cohort	Selected pituitary surgery; total resection 26/29, near-total 1/29, subtotal 2/29.	Visual outcomes were not the primary focus. Compared with age 65–74, patients ≥ 75 had more preoperative TSH-response deficiency (20.7% vs. 4.3%; *p* = 0.017), postoperative TSH-response deficiency (55.2% vs. 32.9%; *p* = 0.045), and low free T3 secretion (48.3% vs. 21.4%; *p* = 0.015).	Median stay 14 days and median discharge mRS 0, similar to the younger comparison group.	Age-related thyroid physiology complicates endocrine interpretation. Only ASA I–II patients were included, and immediate postoperative testing may not represent durable function.
Garvayo et al., 2023 Pituitary adenoma/PitNET [3]	Age ≥ 75; *n* = 155; median age 78; single expert-team cohort	Standard or extended endonasal surgery for visual compression and selected secreting tumors; >80% resection in 77%.	Among 123 with visual deficits, 82 (67%) improved and 41 remained stable; none worsened. Normal pituitary function recovered in 10/95 (11%); new anterior deficit 3/60 (5%); transient DI 5.8%, persistent DI 0%, transient SIADH 6%.	Immediate complications 8/155 (5%); 30-day mortality 0.6%; CSF leak 1.9%; meningitis 0.6%; epistaxis 1.3%. Single-surgery control 97%; control 97.3% at 2 years and 86.2% at 5 years. Extended transtuberculum approach predicted complications: OR 13.77 (95% CI 1.77–107.17; *p* = 0.012); age and ASA III were not significant.	Standard transsellar surgery provided durable decompression with low morbidity; complex extended approaches carried disproportionate risk.
Gould et al., 2025 Craniopharyngioma [23]	SEER 2000–2022; age ≥ 80; *n* = 74	No resection 58.1%; STR 32.4%; GTR 9.5%; radiotherapy 9.5%.	Visual, hypothalamic, endocrine, functional, and quality-of-life outcomes were unavailable.	Mean OS: 42.7 months without resection, 26.1 after STR, and 67.9 after GTR; overall Kaplan–Meier *p* = 0.37. STR versus no resection HR 2.207 (95% CI 1.058–4.605; *p* = 0.035); GTR HR 1.069 (*p* = 0.891); radiotherapy HR 1.252 (*p* = 0.657).	No treatment benefit was established. The adverse STR association is highly vulnerable to confounding by anatomy, treatment intent, and selection.
Gould et al., 2025 Pituitary adenoma [24]	SEER 2000–2022; age ≥ 85, *n* = 686; age 75–84, *n* = 2571	Among patients ≥ 85, 11.8% underwent resection: STR 10.8% and GTR 1.0%.	Visual, endocrine, functional, and perioperative outcomes were unavailable.	Age ≥ 85, mean OS: 23.7 months without resection, 32.9 after STR, and 39.3 after GTR. STR HR 0.557 (95% CI 0.367–0.845; *p* = 0.006); GTR HR 0.735 (95% CI 0.272–1.985; *p* = 0.544); tumor size HR 1.004 (95% CI 1.001–1.007; *p* = 0.005).	STR was associated with longer OS, but severe selection bias and absent clinical outcomes preclude causal interpretation.
**Panel B. Spinal and primary axial tumors: neurologic recovery and survival evidence (3 reports)**					
**Study/Disease**	**Age-Specific Population and Source**	**Treatment Strategy/Indication**	**Functional or Tumor-Specific Outcome**	**Safety, Tumor Control, and Adjusted Findings**	**Principal Interpretation/Limitation**
Lenga et al., 2022 Spinal meningioma [4]	Age ≥ 80; *n* = 30; predominantly thoracic intradural extramedullary tumors; single-center surgical cohort	Posterior decompression and microsurgical resection; Simpson II in 25/30 and III in 5/30.	Mean motor score improved from 85.9+/−12.3 to 93.6+/−8.3; modified McCormick score improved from 3.2+/−1.4 to 1.8+/−0.9; both *p* < 0.001.	In-hospital mortality 6.7%; 90-day mortality 10.0%; 30-day readmission 10.0%. Age-adjusted CCI predicted complications: OR 2.1 (95% CI 1.1–4.3; *p* = 0.004). No surgery for recurrence or secondary instability over 2 years.	Direct evidence of neurologic and ambulatory recovery, offset by meaningful systemic morbidity and early mortality; no nonoperative comparator.
Singh et al., 2026 Spinal/axial osteosarcoma [76]	SEER 17, 2000–2022; age ≥ 75; *n* = 117; grade-available subgroup *n* = 44; vertebral-column and sacrum/pelvis codes	No resection 91/117; STR 21/117; GTR 5/117; chemotherapy 27/117; radiotherapy 38/117.	No neurologic, ambulatory, pain, discharge, or quality-of-life outcomes were available.	Average survival: 10.4 months without resection, 22.7 after STR, and 25.4 after GTR. STR HR 0.499 (95% CI 0.279–0.892; *p* = 0.019); GTR HR 0.563 (95% CI 0.222–0.731; *p* = 0.009). Grade-subgroup treatment estimates were extreme and based on only 44 patients.	Registry associations favored resection and multimodal therapy, but site ambiguity, only five GTRs, absent margins, and severe selection bias prevent causal inference.
Griffith et al., 2026 Spinal ependymoma [9]	NCDB; age ≥ 75; *n* = 126; comparison age 65–74	Surgery in 88/126 (70%) versus 252/296 (85%) at age 65–74 (*p* < 0.001); radiotherapy in 16/126 (13%).	No neurologic, ambulatory, bladder, pain, discharge, or quality-of-life outcomes were available.	Median OS 106.0 months with surgery versus 59.7 without surgery (log-rank *p* = 0.018); surgery HR 0.46 (95% CI 0.24–0.89; *p* = 0.021). Age HR 1.15/year, Charlson–Deyo ≥ 2 HR 4.41, and size HR 1.02/mm; radiotherapy was not significant.	Surgery was associated with longer survival, but EOR, compartment, neurologic morbidity, and 1075 excluded patients with missing data limit interpretation.
**Panel C. Vestibular schwannoma: cranial-nerve, functional, and residual-tumor outcomes (2 reports)**					
**Study/Disease**	**Age-Specific Population and Source**	**Treatment Strategy/Indication**	**Functional or Tumor-Specific Outcome**	**Safety, Tumor Control, and Adjusted Findings**	**Principal Interpretation/Limitation**
Helal et al., 2021 Vestibular schwannoma [5]	Age ≥ 75; *n* = 24, including 8 age ≥ 80; Mayo Clinic surgical cohort; mean tumor diameter 2.76 cm and 3.71 cm in octogenarians	Surgery for large/compressive or progressive tumors, hydrocephalus, disabling symptoms, or failed/declined SRS; GTR 20.8%, NTR 12.5%, STR 66.7%.	HB I–II in 14/24 overall. Among evaluable patients, favorable facial function occurred in 2/7 octogenarians versus 12/16 age 75–79 (*p* = 0.036). No serviceable hearing remained. Nine required out-of-home disposition, but all ultimately returned to independent living.	One postoperative death after acute subdural hematoma; no CSF leak or infection. Tumor control 18/22 (82%); all 4 progressions occurred in octogenarians after STR, with a mean of 34.5 months. Age and size correlated with facial outcome in univariate analysis; ASA and mFI-5 did not predict long-term mRS.	Microsurgery enabled functional rescue in selected patients but carried major facial-nerve, postacute care, and residual-progression tradeoffs.
Lefevre et al., 2024 Vestibular schwannoma [16]	Age ≥ 80; *n* = 13; Paris surgical series; 12/13 Koos IV; mean extrameatal diameter 34.2 mm	Observation followed by surgery for progression, severe imbalance, or trigeminal pain in most patients; preplanned partial resection in 12/13 and GTR in 1/13.	HB I–II immediately and at follow-up in 10/13 (77%); facial nerve anatomically preserved in all. No serviceable ipsilateral hearing remained. Severe imbalance improved in 8/11; 8 discharged home and 5 to rehabilitation.	Three Clavien–Dindo grade III complications and no grade IV–V events. Two recurrences followed partial resection; one later death followed rapid cystic regrowth at 45 months.	Planned partial resection may protect facial and balance function, but hearing was not preserved and residual disease required prolonged surveillance.
**Panel D. Cross-study synthesis**					
**Domain**	**Evidence Summary**		**Interpretation**		**Confidence**
Dominant treatment benefit	Sellar surgery most consistently restored or stabilized vision; spinal meningioma surgery improved motor and ambulatory function; vestibular schwannoma surgery primarily relieved mass effect, imbalance, pain, or hydrocephalus. Registry-based malignant spinal studies measured survival without neurologic outcomes.		Success should be defined by the tumor-specific harm being prevented rather than by a universal survival or resection endpoint.		Moderate
Extent of resection	Adequate decompression and durable control frequently occurred without GTR in pituitary adenoma and vestibular schwannoma. Spinal malignant-tumor registry associations could not account for resectability or margins.		Maximal safe, function-oriented surgery is more defensible than anatomical completeness when additional resection threatens neurologic or endocrine function.		Moderate
Functional and physiologic selection	Expert-center sellar cohorts selected patients by operative fitness and anatomy; CCI predicted spinal meningioma complications; no validated frailty threshold existed for vestibular schwannoma.		Chronological age should not serve as the sole inclusion or exclusion criterion, but direct assessment of reserve and recovery capacity is essential.		Low–moderate
Residual-disease pathway	Residual pituitary tumors were often controlled, whereas subtotal vestibular schwannoma resection produced later progression and one tumor-related death; salvage SRS or reoperation was sometimes required.		Planned residual disease is a longitudinal strategy requiring surveillance and predefined salvage options.		Moderate
Evidence limitations	No prospective comparative study was identified. Registry reports lacked function, complications, endocrine outcomes, margins, and treatment intent; institutional cohorts were small and highly selected.		The available evidence supports structured selection and counseling but does not establish causal treatment effects or universal operative thresholds.		High

Abbreviations: ASA, American Society of Anesthesiologists; CCI, Charlson Comorbidity Index; CI, confidence interval; CSF, cerebrospinal fluid; DI, diabetes insipidus; EOR, extent of resection; GTR, gross-total resection; HB, House–Brackmann; HR, hazard ratio; KPS, Karnofsky Performance Status; mFI-5, 5-item modified frailty index; mRS, modified Rankin Scale; NCDB, National Cancer Database; NTR, near-total resection; OR, odds ratio; OS, overall survival; PitNET, pituitary neuroendocrine tumor; RT, radiotherapy; SEER, Surveillance, Epidemiology, and End Results; SIADH, syndrome of inappropriate antidiuretic hormone secretion; SRS, stereotactic radiosurgery; STR, subtotal resection; TSH, thyroid-stimulating hormone;. Notes: All findings are report-level and observational. No pooled effect estimate was calculated because diseases, treatment intent, interventions, and outcome definitions were heterogeneous. Registry associations should not be interpreted causally. The spinal/axial osteosarcoma cohort included vertebral-column and combined sacrum/pelvis topography codes. Descriptive visual aggregation reported in the text is not a meta-analysis.

**Table 5:** Evidence-informed cross-tumor oncogeriatric framework for neurosurgical intervention in adults aged 75 years or older. Structured synthesis of the 80-report corpus; not a validated clinical prediction rule.

**Panel A. Universal oncogeriatric decision sequence**				
**Step**	**Core Question**		**Required Assessment**	**Decision Output**
**1. Define urgency and intent**	What irreversible harm is expected without intervention, and is the goal survival extension, neurologic rescue, symptom relief, tissue diagnosis, or local control?		Natural history and growth; grade or molecular biology; symptom severity; neurologic trajectory; need for tissue.	A clearly stated, tumor-specific treatment goal.
**2. Establish physiologic and functional reserve**	Can the patient survive the intervention and recover to a state compatible with the treatment goal?		KPS, ECOG, or mRS; ASA and comorbidity; frailty; cognition; nutrition; baseline independence; social and caregiver support.	An individualized estimate of perioperative and recovery capacity.
**3. Estimate achievable surgical benefit**	What decompression, resection, cytoreduction, or diagnosis can realistically be achieved without unacceptable neurologic injury?		Tumor size and anatomy; eloquence; multifocality; approach complexity; resectability; surgeon and center expertise.	A predefined operative endpoint based on achievable rather than theoretical benefit.
**4. Model postoperative pathway feasibility**	Will function be preserved sufficiently to permit the next required treatment step?		Expected functional change; complication and non-home-discharge risk; rehabilitation capacity; RT/TMZ tolerance; surveillance and salvage access.	A longitudinal plan incorporating adjuvant therapy, rehabilitation, surveillance, or salvage.
**5. Compare lower-burden alternatives**	Can observation, biopsy, stereotactic radiosurgery, conventional radiotherapy, or medical therapy achieve the goal with less burden?		Alternative efficacy; time to benefit; toxicity; symptom urgency; need for tissue; feasibility of delayed intervention.	A comparative net-benefit judgment rather than a surgery-versus-no-surgery default.
**6. Align with remaining-life priorities**	Will the anticipated benefit occur within the patient’s remaining-life horizon and match the outcomes the patient values?		Competing life expectancy; time at home; caregiver burden; acceptable disability; communication needs; patient goals.	A goal-concordant recommendation.
**Panel B. Tumor-specific definition of benefit and operative success**				
**Disease Setting**	**Dominant Treatment Goal**	**Preferred Operative Endpoint**	**Clinically Meaningful Success**	**Principal Tradeoff or Pathway Consideration**
**Glioma/glioblastoma**	Extend survival while preserving access to multimodal therapy.	Maximal safe resection when meaningful cytoreduction is achievable without disrupting function.	Overall survival accompanied by preserved postoperative function, home discharge, and initiation or completion of RT/TMZ.	Postoperative deficit, complication, or KPS decline may eliminate the survival opportunity by preventing adjuvant therapy.
**Presumed benign intracranial meningioma**	Relieve symptoms and maintain or restore independence.	Maximal safe, symptom-directed resection rather than anatomical completeness at any cost.	Improved or stable KPS/mRS, home discharge, symptom relief, and durable local control.	New neurologic deficit, systemic complication, rehabilitation dependence, and competing mortality may outweigh additional resection.
**Atypical or high-grade meningioma**	Achieve oncologic control without sacrificing independence.	Gross-total resection when safely achievable; otherwise, planned residual disease with a defined adjuvant strategy.	Preserved function together with local control, PFS, and meningioma-specific survival.	Potential oncologic value of greater resection or RT must be balanced against functional injury and remaining-life horizon.
**Pituitary adenoma/PitNET**	Restore vision and obtain durable tumor control.	Adequate chiasmal decompression while preserving pituitary and neurovascular structures.	Visual improvement or stability, low permanent endocrine morbidity, and durable control.	Endocrine recovery is uncommon, and extended skull-base approaches carry disproportionate risk.
**Craniopharyngioma**	Relieve selected symptoms while limiting hypothalamic and endocrine burden.	Symptom-directed intervention rather than routine pursuit of maximal resection.	Visual or mass-effect improvement with acceptable endocrine, hypothalamic, and treatment burden.	Registry survival associations do not establish a resection-extent benefit in this population.
**Spinal meningioma**	Reverse progressive myelopathy and preserve ambulation.	Safe decompression and resection sufficient to relieve spinal cord compression.	Improved motor function, ambulation, independence, and acceptable 90-day morbidity.	Systemic complications and comorbidity-related early mortality remain clinically important.
**Malignant spinal/primary axial tumors**	Preserve neurologic function and achieve disease-appropriate oncologic control.	Disease-specific resection with neurologic or margin-aware stopping rules within a multidisciplinary pathway.	Neurologic preservation together with PFS or survival, pain control, and acceptable treatment toxicity.	Registry survival associations lack detailed neurologic outcomes, margins, operative morbidity, and treatment-selection data.
**Vestibular schwannoma**	Provide functional rescue when observation or SRS cannot address mass effect or severe symptoms.	Planned partial or subtotal decompression when further dissection threatens facial-nerve function.	Relief of imbalance, pain, hydrocephalus, or brainstem compression with preserved facial function and independence.	Hearing preservation was not demonstrated, and residual disease requires long-term surveillance and salvage planning.
**Other primary tumors**	Define individualized goals according to tumor biology and anatomy.	No universal operative endpoint.	Disease-specific function, symptom control, survival, and treatment burden.	Sparse heterogeneous evidence precludes a cross-diagnosis treatment-effect conclusion.

Note: The framework synthesizes observational evidence and is intended to structure tumor-specific, goal-concordant decision-making. It should not be interpreted as a validated clinical prediction rule or as evidence that surgery is superior to lower-burden alternatives. Abbreviations: ASA, American Society of Anesthesiologists; ECOG, Eastern Cooperative Oncology Group; GTR, gross-total resection; KPS, Karnofsky Performance Status; MGMT, O6-methylguanine-DNA methyltransferase; mRS, modified Rankin Scale; PFS, progression-free survival; PitNET, pituitary neuroendocrine tumor; RT, radiotherapy; SRS, stereotactic radiosurgery; TMZ, temozolomide.

## Data Availability

The data supporting the findings of this scoping review were derived from the published literature cited in the manuscript. Data extracted from the included studies are provided within the article and its Supplementary Materials. No additional datasets were generated during this study.

## Supplementary Materials

The following supporting information can be downloaded at https://www.mdpi.com/article/10.3390/curroncol33090550/s1, Table S1: Complete database search strategies, search dates, and retrieval counts; Table S2: Characteristics of included reports [1,2,3,4,5,6,7,8,9,10,11,12,13,14,15,16,17,18,19,20,21,22,23,24,26,27,28,29,30,31,32,33,34,35,36,37,38,39,40,41,42,43,44,45,46,47,48,49,50,51,52,53,54,55,56,57,58,59,60,61,62,63,64,65,66,67,68,69,70,71,72,73,74,75,76,77,78,79,80,81].
