# Supplementary material for: Beyond Chronological Age: A Scoping Review of Neurosurgical Intervention for Central Nervous System Tumors in the Very Elderly
Source: Curr Oncol. 2026 Sep 10;33(9):550. doi: 10.3390/curroncol33090550 (PMC13605894; doi:10.3390/curroncol33090550)
Supplement: Supplementary file 1 [file curroncol-33-00550-s001.zip › curroncol-4509021-supplementary.pdf]

Supplementary Table S1. Complete Database Search Strategies, Search Dates, and Retrieval Counts

Database	Date Searched	Retrieval Counts	Search String
**PubMed/Medline**	July 24th, 2026	119	(<br>"very elderly"[tiab]<br>OR "oldest old"[tiab]<br>OR "oldest-old"[tiab]<br>OR octogenarian*[tiab]<br>OR nonagenarian*[tiab]<br>OR "75 years or older"[tiab]<br>OR "75 years and older"[tiab]<br>OR "aged 75 years"[tiab]<br>OR "age 75 years"[tiab]<br>OR "80 years or older"[tiab]<br>OR "80 years and older"[tiab]<br>OR "aged 80 years"[tiab]<br>OR "age 80 years"[tiab]<br>OR "85 years or older"[tiab]<br>OR "85 years and older"[tiab]<br>OR "aged 85 years"[tiab]<br>OR "age 85 years"[tiab]<br>OR "90 years or older"[tiab]<br>OR "90 years and older"[tiab]<br>OR "aged 90 years"[tiab]<br>OR "age 90 years"[tiab]<br>)<br>AND<br>(<br>glioblastoma*[tiab]<br>OR glioma*[tiab]<br>OR astrocytoma*[tiab]<br>OR oligodendroglioma*[tiab]<br>OR meningioma*[tiab]<br>OR ependymoma*[tiab]<br>OR schwannoma*[tiab]<br>OR neurilemmoma*[tiab]<br>OR "pituitary adenoma"[tiab]<br>OR "pituitary adenomas"[tiab]<br>OR craniopharyngioma*[tiab]<br>OR chordoma*[tiab]<br>OR osteosarcoma*[tiab]<br>OR hemangioblastoma*[tiab]<br>OR "brain tumor"[tiab]<br>OR "brain tumors"[tiab]<br>OR "brain tumour"[tiab]<br>OR "brain tumours"[tiab]<br>OR "intracranial tumor"[tiab]<br>OR "intracranial tumors"[tiab]<br>OR "intracranial tumour"[tiab]<br>OR "intracranial tumours"[tiab]<br>OR "skull base tumor"[tiab]

			OR "skull base tumors"[tiab]<br>OR "skull base tumour"[tiab]<br>OR "skull base tumours"[tiab]<br>OR "spinal tumor"[tiab]<br>OR "spinal tumors"[tiab]<br>OR "spinal tumour"[tiab]<br>OR "spinal tumours"[tiab]<br>OR "spinal cord tumor"[tiab]<br>OR "spinal cord tumors"[tiab]<br>OR "spinal cord tumour"[tiab]<br>OR "spinal cord tumours"[tiab]<br>)<br>AND<br>(<br>neurosurg*[tiab]<br>OR surg*[tiab]<br>OR operat*[tiab]<br>OR resect*[tiab]<br>OR biops*[tiab]<br>OR craniotom*[tiab]<br>OR transsphenoidal[tiab]<br>OR endonasal[tiab]<br>OR laminectom*[tiab]<br>OR laminoplast*[tiab]<br>OR decompress*[tiab]<br>)<br>NOT<br>(<br>animals[mh] NOT humans[mh]<br>)
**Embase**	July 25th, 2026	189	(("very elderly" or "oldest old" or "oldest-old" or octogenarian* or nonagenarian* or "75 years or older" or "75 years and older" or "aged 75 years" or "age 75 years" or "80 years or older" or "80 years and older" or "aged 80 years" or "age 80 years" or "85 years or older" or "85 years and older" or "aged 85 years" or "age 85 years" or "90 years or older" or "90 years and older" or "aged 90 years" or "age 90 years") and (glioblastoma* or glioma* or astrocytoma* or oligodendroglioma* or meningioma* or ependymoma* or schwannoma* or neurilemmoma* or "pituitary adenoma" or "pituitary adenomas" or craniopharyngioma* or chordoma* or osteosarcoma* or hemangioblastoma* or "brain tumor" or "brain tumors" or "brain tumour" or "brain tumours" or "intracranial tumor" or "intracranial tumors" or "intracranial tumour" or "intracranial tumours" or "skull base tumor" or "skull

			base tumors" or "skull base tumour" or "skull base tumours" or "spinal tumor" or "spinal tumors" or "spinal tumour" or "spinal tumours" or "spinal cord tumor" or "spinal cord tumors" or "spinal cord tumour" or "spinal cord tumours") and (neurosurg* or surg* or operat* or resect* or biops* or craniotom* or transsphenoidal or endonasal or laminectom* or laminoplast* or decompress*)).ti,ab,kw.
**Scopus**	July 25th, 2026	87	TITLE-ABS ({very elderly} OR {oldest old} OR {oldest-old} OR octogenarian* OR nonagenarian* OR {75 years or older} OR {75 years and older} OR {aged 75 years} OR {age 75 years} OR {80 years or older} OR {80 years and older} OR {aged 80 years} OR {age 80 years} OR {85 years or older} OR {85 years and older} OR {aged 85 years} OR {age 85 years} OR {90 years or older} OR {90 years and older} OR {aged 90 years} OR {age 90 years}) AND TITLE-ABS (glioblastoma* OR glioma* OR astrocytoma* OR oligodendroglioma* OR meningioma* OR ependymoma* OR schwannoma* OR neurilemmoma* OR {pituitary adenoma} OR {pituitary adenomas} OR craniopharyngioma* OR chordoma* OR osteosarcoma* OR hemangioblastoma* OR {brain tumor} OR {brain tumors} OR {brain tumour} OR {brain tumours} OR {intracranial tumor} OR {intracranial tumors} OR {intracranial tumour} OR {intracranial tumours} OR {skull base tumor} OR {skull base tumors} OR {skull base tumour} OR {skull base tumours} OR {spinal tumor} OR {spinal tumors} OR {spinal tumour} OR {spinal tumours} OR {spinal cord tumor} OR {spinal cord tumors} OR {spinal cord tumour} OR {spinal cord tumours}) AND TITLE-ABS (neurosurg* OR surg* OR operat* OR resect* OR biops* OR craniotom* OR transsphenoidal OR endonasal OR laminectom* OR laminoplast* OR decompress*)
**Web of Science**	July 25th, 2026	130	"very elderly"<br>OR "oldest old"<br>OR "oldest-old"<br>OR octogenarian*<br>OR nonagenarian*<br>OR "75 years or older"<br>OR "75 years and older"

	OR "aged 75 years"<br>OR "age 75 years"<br>OR "80 years or older"<br>OR "80 years and older"<br>OR "aged 80 years"<br>OR "age 80 years"<br>OR "85 years or older"<br>OR "85 years and older"<br>OR "aged 85 years"<br>OR "age 85 years"<br>OR "90 years or older"<br>OR "90 years and older"<br>OR "aged 90 years"<br>OR "age 90 years"
	glioblastoma*<br>OR glioma*<br>OR astrocytoma*<br>OR oligodendroglioma*<br>OR meningioma*<br>OR ependymoma*<br>OR schwannoma*<br>OR neurilemmoma*<br>OR "pituitary adenoma"<br>OR "pituitary adenomas"<br>OR craniopharyngioma*<br>OR chordoma*<br>OR osteosarcoma*<br>OR hemangioblastoma*<br>OR "brain tumor"<br>OR "brain tumors"<br>OR "brain tumour"<br>OR "brain tumours"<br>OR "intracranial tumor"<br>OR "intracranial tumors"<br>OR "intracranial tumour"<br>OR "intracranial tumours"<br>OR "skull base tumor"<br>OR "skull base tumors"<br>OR "skull base tumour"<br>OR "skull base tumours"<br>OR "spinal tumor"<br>OR "spinal tumors"<br>OR "spinal tumour"<br>OR "spinal tumours"<br>OR "spinal cord tumor"<br>OR "spinal cord tumors"<br>OR "spinal cord tumour"<br>OR "spinal cord tumours"

	neurosurg*<br>OR surg*<br>OR operat*<br>OR resect*<br>OR biops*<br>OR craniotom*<br>OR transsphenoidal<br>OR endonasal<br>OR laminectom*<br>OR laminoplast*<br>OR decompress*

Supplementary Table S2. Characteristics of Included Reports

Reference	Study Title	Year	Tumor category	Study design	Setting / data source	Study period	Population / age criterion / N	Primary exposure / comparator	Follow-up	Funding
[1]	Patterns of care and outcomes among elderly individuals with primary malignant astrocytoma.	2008	Glioma/glioblastoma	Retrospective database/registry analysis	SEER-Medicare; Population cancer registry	1991–1999	AA/GBM; age ≥66 overall (GBM n=1,753; AA n=205); very-elderly GBM subgroup age ≥75 analyzed; subgroup n not reported in abstract	Biopsy/resection ± radiotherapy; treatment combinations compared across age strata	Medicare coverage required for 12 months after diagnosis	Author support: National Cancer Institute grants K07-CA91849 (J.S. Barnholtz-Sloan) and K08-CA101954 (A.E. Sloan).
[26]	Surgery for high-grade gliomas in the aging.	2013	Glioma/glioblastoma	Prospective observational cohort	Oslo and Haukeland University Hospitals; multicenter clinical cohort	2008–2009	WHO grade 3/4 glioma; age ≥60; n=80 (age range 60–83); age ≥80 analyzed; ≥75 subgroup n not reported	Craniotomy/resection for HGG; adjuvant radiochemotherapy assessed	6-month clinical assessment; mortality evaluated at 2 years	No institutional, private, or corporate financial support for this study.
[17]	A Surveillance, Epidemiology and End Results-Medicare data analysis of elderly patients with glioblastoma multiforme:	2015	Glioma/glioblastoma	Retrospective database/registry analysis	SEER-Medicare; Population cancer registry	1997–2009	GBM; age >66; n=3,759 (range 66–97); ≥75 subgroup n not reported in abstract	Diagnostic surgery (resection or biopsy), followed by no treatment vs standard/hypofractionated RT ± TMZ or TMZ alone	NR	Not reported

	Treatment patterns, outcomes and cost.									
[27]	Factors associated with increased survival after surgical resection of glioblastoma in octogenarians.	2015	Glioma/glioblastoma	Retrospective clinical cohort/series	Institutional clinical cohort; center count not stated	NR	GBM; age ≥80; n=58	Tumor-directed surgery; no adjuvant therapy vs radiotherapy vs chemoradiation	NR	No support or funding reported.
[28]	Patient and treatment factors associated with survival among adult glioblastoma patients: A USA population-based study from 2000-2010.	2015	Glioma/glioblastoma	Retrospective database/registry analysis	SEER; Population cancer registry	2000–2009	Adult GBM; n=14,675; age >75 included as prognostic stratum; age >75 subgroup n not reported	Biopsy/partial resection vs gross-total resection; radiotherapy exposure also assessed	Minimum follow-up 12 months	Not reported
[29]	Glioblastoma multiforme in the very elderly.	2016	Glioma/glioblastoma	Mixed prospective-retrospective cohort	Two Australian hospitals; ACCORD/BioGrid clinical database	2008–2011	GBM; age ≥80; n=40	Surgery (extent of resection reported) ± postoperative radiotherapy/chemotherapy; no formal nonoperative comparator	NR	Not reported
[30]	Survival Trends in Elderly Patients with Glioblastoma in the United States: a Population-based Study.	2016	Glioma/glioblastoma	Retrospective database/registry analysis	SEER; Population cancer registry	1999–2010	GBM; age ≥70; n=5,575; ≥75 subgroup n not separately reported in abstract	Population-level surgery/resection and adjuvant-treatment patterns; pre-Stupp vs Stupp-era survival	NR	None.
[31]	Gliosarcoma in septuagenarians and	2017	Glioma/glioblastoma	Retrospective database/registry analysis	NCDB; National clinical/administrative database	NR	Resected gliosarcoma in	All underwent resection; adjuvant chemoradiation vs radiotherapy alone	1-year OS reported; follow-up	No specific grant from funding agencies in the

	octogenarians: What is the impact of adjuvant chemoradiation?						septuagenarians and octogenarians; total n=210; ≥75 subgroup n not separately reported in abstract		duration NR	public, commercial, or not-for-profit sectors.
[32]	Survival Outcomes of Elderly Patients With Glioblastoma Multiforme in Their 75th Year or Older Treated With Adjuvant Therapy.	2017	Glioma/glioblastoma	Retrospective analysis of prospectively maintained database	Institutional clinical cohort; center count not stated	2007–2015	GBM; age ≥75 receiving active treatment; n=73	Active multimodal treatment; temozolomide vs none and treatment completion assessed	NR	Not reported
[33]	Characteristics and outcomes of elderly patients with diffuse gliomas: a multi-institutional cohort study by Kansai Molecular Diagnosis Network for CNS Tumors.	2018	Glioma/glioblastoma	Retrospective clinical cohort/series	Kansai Molecular Diagnosis Network for CNS Tumors; multicenter clinical cohort	NR	Primary diffuse glioma; age ≥70; n=140 (median age 75); ≥75 subgroup n not separately reported in abstract	Biopsy/partial/STR/GTR followed by adjuvant RT + TMZ as applicable	NR	Not reported
[34]	Management and Outcomes in the Oldest-Old Population with Glioblastoma.	2018	Glioma/glioblastoma	Retrospective clinical cohort/series	Institutional clinical cohort; center count not stated	2004–2009	GBM; age ≥80; n=48	Resection + RT/chemoradiation vs biopsy + RT vs biopsy + best supportive care	NR	Gerry and Nancy Pencer Brain Tumor Center.

[6]	Surgical treatment of glioblastoma in the elderly: the impact of complications.	2018	Glioma/glioblastoma	Retrospective clinical cohort/series	Institutional clinical cohort; center count not stated	1997–2016	GBM; age ≥75; n=82	No surgery vs biopsy vs STR vs GTR	NR	Not reported
[35]	Phase II study of hypofractionated radiation therapy in elderly patients with newly diagnosed glioblastoma with poor prognosis	2018	Glioma/glioblastoma	Prospective single-arm phase II study	Humanitas Cancer Center and Research Hospital, Italy; single-arm phase II cohort	NR	Newly diagnosed GBM; age ≥70; n=30 (median age 75); ≥75 subgroup n not established from the abstract	Postoperative hypofractionated RT (52.5 Gy/15 fractions) ± concurrent/adjuvant TMZ; single-arm phase II	NR	No specific grant from any funding agency in the public, commercial, or not-for-profit sectors.
[12]	To resect or not to resect? Risks and benefits of surgery in older patients with glioblastoma.	2020	Glioma/glioblastoma	Retrospective clinical cohort/series	Single-center clinical cohort	2006–2014	GBM; age 75-84 subgroup; n=85	GTR or STR vs stereotactic biopsy	NR	Not reported
[36]	Competing risk model to determine the prognostic factors and treatment strategies for elderly patients with glioblastoma.	2021	Glioma/glioblastoma	Retrospective database/registry analysis	SEER; Population cancer registry	NR	Histologically confirmed GBM; age ≥65; n=4,975; age ≥75 included as prognostic stratum; stratum n not reported	Extent of resection (including GTR) and RT/chemotherapy/chemoradiotherapy treatment strategies	3-, 6-, and 12-month mortality modeled; follow-up duration NR	National Natural Science Foundation of China (82073893, 81703622, 81472693, 81873635); China Postdoctoral Science Foundation (2018M633002); Hunan Provincial

										Natural Science Foundation of China (2018JJ3838, 2018SK2101, 2020JJ8111); Hunan Provincial Health and Health Committee Foundation of China (C2019186).
[37]	Elderly patients aged over 75 years with glioblastoma: Preoperative status and surgical strategies	2021	Glioma/glioblastoma	Retrospective clinical cohort/series	Institutional clinical cohort; center count not stated	NR	GBM; age ≥75; n=31 (resection 20, biopsy 11)	Resection vs biopsy	NR	Not reported
[18]	Extent of resection and survival outcomes of geriatric patients with glioblastoma: Is there benefit from aggressive surgery?	2021	Glioma/glioblastoma	Retrospective database/registry analysis	SEER; Population cancer registry	2000–2016	GBM; SEER 2000-2016; total n=17,820; age-specific analyses for ≥75 and octogenarians (octogenarian n=3,250)	Biopsy/local excision vs STR vs GTR vs supratotal resection	NR	No funding to disclose.
[38]	Predictors of survival in elderly patients undergoing surgery for glioblastoma	2021	Glioma/glioblastoma	Retrospective clinical cohort/series	Single-center clinical cohort	2005–2018	Surgically treated newly diagnosed GBM; age ≥65; n=150; age ≥75 subgroup	Surgery for newly diagnosed GBM; extent of resection, complications, and adjuvant treatment assessed; age ≥75 vs 65–74	NR	None declared.

							analyzed; subgroup n not reported in abstract			
[39]	Real-world evaluation of the impact of radiotherapy and chemotherapy in elderly patients with glioblastoma based on age and performance status	2021	Glioma/glioblastoma	Retrospective database/registry analysis	NCDB; National clinical/administrative database	2004–2015	GBM; NCDB age ≥80 with KPS ≥70 subgroup; n=223	NCDB treatment combinations (surgery/resection, radiotherapy, chemotherapy) within age ≥80/KPS ≥70 subgroup	median follow-up of 6.8 months (range, 0-151 months)	University of Texas MD Anderson Cancer Center Support Grant P30CA01667; University of Texas MD Anderson Cancer Center Division of Radiation Oncology, Radiation Oncology Strategic Research Initiative grant.
[13]	Do elderly patients (≥ 75 years old) with glioblastoma benefit from more radical surgeries in the era of temozolomide?	2022	Glioma/glioblastoma	Retrospective database/registry analysis	SEER; Population cancer registry	2014–2018 (institutional); 2005–2016 (SEER)	Institutional cohort: GBM, age ≥75, n=50; SEER cohort: GBM, age ≥75, n=6,079	Institutional: partial vs STR vs GTR; SEER: no surgery vs biopsy vs subtotal/partial resection vs GTR	NR	Not reported
[40]	Outcome of glioblastoma resection in patients 80 years of age and older.	2022	Glioma/glioblastoma	Retrospective clinical cohort/series	Single-center clinical cohort	2007–2018	GBM; age ≥80; n=117 (resection 57, biopsy 60)	Resection vs biopsy	NR	Not reported

[41]	Refined efficacy and outcome estimates of surgical treatment in oldest-old patients with glioblastomas based on competing risk model and conditional survival analysis: A surveillance, epidemiology, and end results population-based study.	2022	Glioma/glioblastoma	Retrospective database/registry analysis	SEER; Population cancer registry	2005–2016	GBM; age ≥80; n=3,309	No surgery, biopsy/partial, STR, and GTR; radical (STR/GTR) vs nonradical	NR	No external funding for this project.
[10]	Sarcopenia Diagnosed Using Masseter Muscle Diameter as a Survival Correlate in Elderly Patients with Glioblastoma.	2022	Glioma/glioblastoma	Retrospective clinical cohort/series	Single-center clinical cohort	NR	GBM; age ≥79; n=110	Surgery/resection plus RT and/or TMZ; postoperative functional change and treatment receipt assessed	NR	Not reported
[42]	Outcomes and Treatment Algorithm in Glioblastoma Patients 80 Years and Older.	2023	Glioma/glioblastoma	Retrospective clinical cohort/series	Single-center clinical cohort	2009–2020	GBM; age ≥80; n=123	Surgery/biopsy plus temozolomide and/or radiotherapy; multimodal treatment pathways compared	NR	Not reported

[43]	Treatment benefit in patients aged 80 years or older with biopsy-proven and non-resected glioblastoma is dependent on MGMT promoter methylation status.	2023	Glioma/glioblastoma	Retrospective clinical cohort/series	Institutional clinical cohort; center count not stated	2010–2022	Biopsy-proven IDH-wildtype GBM; age ≥80; n=76	Biopsy-proven nonresected GBM; active treatment vs best supportive care, stratified by MGMT	NR	No funding to report; open-access funding enabled and organized by Projekt DEAL.
[44]	Treatment of very elderly glioblastoma patients ≥ 75 years of age: whom to treat.	2023	Glioma/glioblastoma	Retrospective clinical cohort/series	Single-center clinical cohort	2010–2018	GBM; age ≥75; n=143	Surgery/resection, radiotherapy, and chemotherapy exposures assessed	NR	K.J.W. funded by the Mildred Scheel Career Center Frankfurt (Deutsche Krebshilfe); open-access funding enabled and organized by Projekt DEAL.
[45]	A novel scoring system proposal to guide surgical treatment indications for high grade gliomas in elderly patients: DAK-75.	2024	Glioma/glioblastoma	Retrospective clinical cohort/series	Single-center clinical cohort	NR	High-grade glioma; age ≥75; n=102	Surgical selection (resection vs no resection) analyzed through DAK-75 prognostic model	NR	Not reported
[46]	Geriatric grade 2 and 3 gliomas: A national cancer database analysis of demographics,	2024	Glioma/glioblastoma	Retrospective database/registry analysis	NCDB; National clinical/administrative database	2010–2017	Grade 2/3 intracranial glioma; age 60–89; n=6,257;	Surgery vs nonsurgical management; chemotherapy and radiotherapy utilization also assessed	NR	No specific grant from funding agencies in the public, commercial, or

	treatment utilization, and survival.						octogenarians n=679; ≥75 total n not separately reported			not-for-profit sectors.
[47]	Glioblastoma in the oldest old: Clinical characteristics, therapy, and outcome in patients aged 80 years and older.	2024	Glioma/glioblastoma	Retrospective clinical cohort/series	Multicenter clinical cohort	NR	GBM; age ≥80; n=107 across 6 centers	Surgical strategy plus postoperative therapy; receipt of any postoperative therapy assessed	NR	Not reported
[21]	Surgical resection of glioblastoma in the very elderly: An analysis of survival outcomes using the surveillance, epidemiology, and end results database.	2024	Glioma/glioblastoma	Retrospective database/registry analysis	SEER; Population cancer registry	2000–2020	GBM; age ≥80; n=1,868	Biopsy vs STR vs GTR	NR	Not reported
[48]	Survival outcomes and prognostic factors of infratentorial glioblastoma in the elderly.	2024	Glioma/glioblastoma	Retrospective database/registry analysis	SEER; Population cancer registry	2000–2016	Infratentorial GBM; total n=420; elderly age >65 n=137; age ≥80 subgroup analyzed; ≥75 subgroup n not separately reported	GTR/resection and adjuvant chemotherapy/radiotherapy; elderly vs younger adults and location-specific analyses	NR	Author support: Ankush Chandra and Bryan Ryba were supported by the Howard Hughes Medical Institute (HHMI).

[22]	Clinical Predictors of Overall Survival in Very Elderly Patients With Glioblastoma: A National Cancer Database Multivariable Analysis.	2025	Glioma/glioblastoma	Retrospective database/registry analysis	NCDB; National clinical/administrative database	1989–2016	GBM; NCDB age ≥80; n=578	GTR/other surgery plus radiotherapy and chemotherapy; readmission also assessed	NR	No funding or financial support.
[49]	Management considerations for very elderly patients with intracranial low-grade gliomas: A surveillance, epidemiology, and end results database multivariable assessment.	2026	Glioma/glioblastoma	Retrospective database/registry analysis	SEER; Population cancer registry	2000–2022	Intracranial low-grade glioma; age ≥85; n=182 (no resection 130, STR 36, GTR 16)	No resection vs STR vs GTR	NR	No financial support for the research, authorship, and/or publication.
[50]	Maximal resection improves survival in MGMT methylated very elderly glioblastoma patients.	2026	Glioma/glioblastoma	Retrospective clinical cohort/series	Institutional clinical cohort; center count not stated	2016–2022	IDH-wildtype GBM; age ≥75; n=108 (biopsy 36, submaximal 30, maximal 42)	Biopsy vs resection; maximal vs submaximal resection	NR	No funds, grants, or other support received during preparation of the manuscript; open-access funding enabled and organized by Projekt DEAL.
[51]	Prognostic Impact of Clinical Diagnosis-based Treatment in	2026	Glioma/glioblastoma	Retrospective clinical cohort/series	Single-center clinical cohort	2011–2025	GBM; age ≥80; n=31 (resection 6, biopsy 14,	Resection, biopsy, and treatment based on clinical diagnosis without surgery	NR	JSPS KAKENHI grant JP24K10399 to Takahiro Ono.

	Glioblastoma Patients Aged ≥80 Years: A Single-center Cohort Study Focused on Performance Status Maintenance.						clinical diagnosis 11)			
[52]	Short and long term post-operative outcomes in elderly versus very elderly glioblastoma patients.	2026	Glioma/glioblastoma	Retrospective clinical cohort/series	Institutional clinical cohort; center count not stated	2008–2020	Surgically treated GBM; age ≥75 n=79 vs age 65-74 n=165	Surgically treated GBM; postoperative RT/TMZ receipt compared by age group	NR	Clinical data coordination/retrieval support from the Core for Clinical Research Data Acquisition, supported in part by the Johns Hopkins Institute for Clinical and Translational Research (UL1TR001079); Khatib Brain Tumor Center support acknowledged; authors received no financial support for authorship and/or publication.
[14]	Survival and function in elderly patients with GBM: the	2026	Glioma/glioblastoma	Retrospective clinical cohort/series	Single-center clinical cohort	2016–2024	Lobar GBM; age ≥80 subgroup n=27	Resection vs biopsy	NR	Northwestern Medicine Pre-med Gap Year and

	role of surgical resection with contemporary multimodal therapy.									Summer Internship Program.
[53]	Intracranial meningiomas in the 9th decade of life: a retrospective study of 17 surgical cases.	1995	Intracranial meningioma	Retrospective clinical cohort/series	Institutional clinical cohort; center count not stated	NR	Intracranial meningioma; age ≥80; n=17	Intracranial meningioma resection; no nonoperative comparator	NR	Not reported
[54]	Intracranial meningioma surgery in the ninth decade of life.	2007	Intracranial meningioma	Retrospective clinical cohort/series	Single-center clinical cohort	1990–2005	Intracranial meningioma; ninth decade; n=74	Intracranial meningioma resection; no nonoperative comparator	median follow-up period was 94 months (range, 15-147 mo)	Not reported
[55]	Surgery for intracranial meningiomas in patients older than 80 years	2007	Intracranial meningioma	Retrospective clinical cohort/series	Single-center clinical cohort	1998–2005	Intracranial meningioma; age >80; n=11	Intracranial meningioma resection; no nonoperative comparator	NR	Not reported
[56]	Treatment and survival of patients with nonmalignant intracranial meningioma: results from the Surveillance, Epidemiology, and End Results	2011	Intracranial meningioma	Retrospective database/registry analysis	SEER; Population cancer registry	2004–2007	SEER 2004-2007; n=12,284; age ≥80 category n=2,132	Resection vs biopsy/no surgery; age-stratified treatment utilization	NR	NIH R01 grants CA109468, CA109461, CA109745, CA108473, and CA109475; Brain Science Foundation; Meningioma Mommas.

	Program of the National Cancer Institute. Clinical article.								
[57]	Clinical features and surgical management of intracranial meningiomas in the elderly.	2017	Intracranial meningioma	Retrospective clinical cohort/series	Institutional clinical cohort; center count not stated	2007–2013	Intracranial meningioma; age ≥75 subgroup n=16	Intracranial meningioma resection; age-group/matched comparison as specified in population	NR	Not reported
[58]	Morbidity and Mortality of Meningioma Resection Increases in Octogenarians.	2018	Intracranial meningioma	Retrospective database/registry analysis	ACS-NSQIP; National clinical/administrative database	2005–2012	Supratentorial meningioma; ACS-NSQIP total n=1,568; age >80 subgroup	Intracranial meningioma resection; age-group/matched comparison as specified in population	NR	Not reported
[59]	Surgical treatment and outcome in patients over 80 years old with intracranial meningioma.	2018	Intracranial meningioma	Retrospective clinical cohort/series	Single-center clinical cohort	2013–2016	Intracranial meningioma; age >80; n=25	Intracranial meningioma resection; no nonoperative comparator	NR	No funding was received for this research.
[2]	Nonmalignant and malignant meningioma incidence and survival in the elderly, 2005-2015, using the Central Brain Tumor Registry of the United States.	2019	Intracranial meningioma	Retrospective database/registry analysis	CBTRUS; Population cancer registry	2005–2015	CBTRUS incidence; SEER survival 2005-2015; age 65+	Treatment utilization and extent of resection/radiotherapy; no direct age-specific treatment-effect comparison	NR	Funding for CBTRUS: Centers for Disease Control and Prevention contract 2016-M-9030; American Brain Tumor Association; The Sontag Foundation; Novocure; AbbVie;

										Musella Foundation; National Cancer Institute contract HHSN26120180017 6P; private and in-kind donations.
[60]	Minimally invasive surgical treatment of intracranial meningiomas in elderly patients (≥ 65 years): outcomes, readmissions, and tumor control.	202 0	Intracranial meningioma	Retrospective clinical cohort/series	Single-center clinical cohort	2008–2019 surgical cohort; 2015–2019 observed comparator	Meningioma surgery cohort: age ≥65, 118 patients/126 operations; age 75–79: 17 operations; age ≥80: 23 operations (40 operations age ≥75); separate observed age ≥65 comparator cohort	Minimally invasive/conventional meningioma resection; younger surgical comparator and separate observed ≥65 cohort	Mean follow-up 36 months (overall surgical cohort)	Support acknowledged from the Pacific Neuroscience Institute Foundation and Saint John's Health Center Foundation.
[61]	Resection of meningiomas in octogenarians: a comparison with a younger geriatric population.	202 0	Intracranial meningioma	Mixed prospective-retrospective cohort	Single-center clinical cohort	1990–2002	Intracranial meningioma; age ≥80 n=49 versus age 70-79 n=272	Intracranial meningioma resection; age-group/matched comparison as specified in population	NR	Not reported
[62]	The Outcome of Intracranial Meningioma Surgery in	202 0	Intracranial meningioma	Retrospective clinical cohort/series	Institutional clinical cohort; center count not stated	2008–2020	Intracranial meningioma; age ≥80 n=31,	Intracranial meningioma resection; age-group/matched comparison as specified in population	NR	Not reported

	Octogenarians: Matched Cohort Study.						matched younger n=31			
[63]	High-grade meningiomas in octogenarian and elderly patients: A population-based SEER analysis.	2021	Intracranial meningioma	Retrospective database/registry analysis	SEER; Population cancer registry	1990–2016	SEER 1990-2016; age ≥80; n=678	Observation vs radiotherapy only vs surgery ± radiotherapy; GTR vs STR among surgical patients	NR	No specific grants from funding agencies.
[64]	Meningioma surgical outcomes and complications in patients aged 75 years and older.	2021	Intracranial meningioma	Retrospective clinical cohort/series	Institutional clinical cohort; center count not stated	1996–2014	Intracranial meningioma; age ≥75; n=103	Intracranial meningioma resection; no nonoperative comparator	Median follow-up was 5.8 years (IQR 1.7-8.7 years)	Linda Wolfe Memorial Meningioma Research Endowment.
[65]	Neurosurgery for intracranial meningioma in patients aged more than 80 years: benefits and rationale.	2021	Intracranial meningioma	Retrospective clinical cohort/series	Single-center clinical cohort	NR	Intracranial meningioma; age >80; n=37	Intracranial meningioma resection; no nonoperative comparator	follow-up was 80.0 months (range: 1-96 months)	Not reported
[66]	Risks and neurological benefits of meningioma surgery in elderly patients compared to young patients.	2021	Intracranial meningioma	Retrospective clinical cohort/series	Institutional clinical cohort; center count not stated	NR	Intracranial meningioma; age ≥80 subgroup within n=768	Intracranial meningioma resection; age-group/matched comparison as specified in population	NR	Open-access funding enabled and organized by Projekt DEAL.
[11]	A nationwide registry study: The 5-factor modified frailty	2022	Intracranial meningioma	Retrospective database/registry analysis	Japanese Diagnosis Procedure Combination database; National	2010–2015	Surgically treated meningioma; Japanese DPC	Intracranial meningioma resection; age-group/matched comparison as specified in population	NR	Japan Society for the Promotion of Science, Grant-in-Aid for Scientific

	index of meningioma surgery in non-elderly and elderly patients.				clinical/administrative database		n=8,138; age ≥75 subgroup			Research (C) (17K10829).
[67]	Case Series of Skull Base Meningioma Resection in the Octogenarian Population.	2022	Intracranial meningioma	Retrospective clinical cohort/series	Institutional clinical cohort; center count not stated	NR	Skull-base meningioma; octogenarians; n=14	Intracranial meningioma resection; no nonoperative comparator	NR	Not reported
[68]	Intracranial Meningiomas in Patients Aged ≥80 Years: Pathological Features and Surgical Problems.	2023	Intracranial meningioma	Retrospective clinical cohort/series	Institutional clinical cohort; center count not stated	NR	Intracranial meningioma; age ≥80 n=17 versus younger groups	Intracranial meningioma resection; age-group/matched comparison as specified in population	NR	Not reported
[15]	Surgery in octogenarians with intracranial meningiomas improves functional outcome at 1 year.	2023	Intracranial meningioma	Retrospective clinical cohort/series	Single-center clinical cohort	2004–2021	Intracranial meningioma; age 80-89; n=30	Intracranial meningioma resection; no nonoperative comparator	NR	Not reported
[69]	Atypical and anaplastic meningiomas in the later decades of life: A national cancer database analysis.	2024	Intracranial meningioma	Retrospective database/registry analysis	NCDB; National clinical/administrative database	2010–2017	NCDB 2010-2017; age 60-89; octogenarians n=911	No surgery vs GTR vs STR; radiotherapy utilization also assessed	NR	No funds, grants, or other support received during preparation of the manuscript.

[7]	Surgical Outcome of Patients With Supratentorial Meningiomas Aged 80 Years or Older-Retrospective International Multicenter Study.	2024	Intracranial meningioma	Retrospective clinical cohort/series	Multicenter clinical cohort	NR	Supratentorial meningioma; age ≥80; n=262	Intracranial meningioma resection; no nonoperative comparator	NR	Study received no funding or financial support; Ilari Rautalin received personal research grants from the Sigrid Juselius Foundation, Finnish Medical Foundation, and Sakari Alhopuro Foundation.
[19]	Surgical Risk in Elderly Patients with Meningiomas in Japan.	2024	Intracranial meningioma	Retrospective database/registry analysis	Japanese Diagnosis Procedure Combination database; National clinical/administrative database	2010–2015	Surgically treated meningioma; Japanese DPC n=8,138; age ≥75 subgroup	Intracranial meningioma resection; age-group/matched comparison as specified in population	NR	Japan Society for the Promotion of Science, Grant-in-Aid for Scientific Research (C) (23K08521).
[20]	Clinical predictors of overall survival in very elderly meningioma patients: a surveillance, epidemiology, and end results (SEER) database analysis.	2025	Intracranial meningioma	Retrospective database/registry analysis	SEER; Population cancer registry	2000–2020	SEER 2000-2020; age ≥80; n=590	No operation vs STR vs GTR; radiotherapy vs none	NR	No funds, grants, or other monetary support received during preparation of the manuscript.
[70]	Optimal treatment regimen for very elderly patients with atypical	2025	Intracranial meningioma	Retrospective database/registry analysis	NCDB; National clinical/administrative database	2004–2019	NCDB 2004-2019; age >80; n=382	GTR vs STR; adjuvant radiotherapy vs no radiotherapy	NR	None disclosed in the Funding section.

	meningioma: an analysis of survival outcomes using the National Cancer Database (NCDB).									
[8]	Surgical outcomes in octogenarian meningioma patients: a multicenter retrospective analysis of frailty and radiological predictors : Frailty in octogenarians undergoing meningioma resection.	2025	Intracranial meningioma	Retrospective clinical cohort/series	Multicenter clinical cohort	2010–2023	Intracranial meningioma; age ≥80; n=189	Intracranial meningioma resection; no nonoperative comparator	NR	No specific grant from any funding agency in the public, commercial, or not-for-profit sectors.
[77]	Brain tumor surgery in geriatric patients: a critical analysis in 44 patients over 80 years.	1999	Mixed/other intracranial tumors	Retrospective clinical cohort/series	Institutional clinical cohort; center count not stated	NR	Primary benign and primary malignant brain tumors; age >80; eligible primary-tumor n=28	Tumor-directed cranial surgery; benign vs primary malignant tumor subgroups	NR	Not reported
[78]	Neoplasms Involving the Central Nervous	2003	Mixed/other intracranial tumors	Retrospective clinical cohort/series	Single-center clinical cohort	1992–2002	Primary CNS neoplasms; age ≥75; n=90	No treatment comparison; descriptive neuropathology/tumor-taxonomy evidence	NR	Not reported

	System in the Older Old									
[79]	Surgical precautions to maintain quality of life in patients aged more than 80 years with benign brain tumors	2005	Mixed/other intracranial tumors	Retrospective clinical cohort/series	Institutional clinical cohort; center count not stated	NR	Benign primary brain tumors; age 80–100; n=11	Surgery for benign primary brain tumors; no comparator	NR	Not reported
[80]	Patterns of Care After Surgery for Elderly Patients With Malignant Brain Tumors: Data from the National Health Insurance Service in South Korea.	2019	Mixed/other intracranial tumors	Retrospective database/registry analysis	Korean National Health Insurance Service; National clinical/administrative database	2008–2016	Primary malignant brain neoplasm; age ≥80; n=130	Postoperative no treatment vs radiotherapy vs chemotherapy vs chemoradiotherapy	NR	Not reported
[81]	Octogenarian Brain Tumor Registry: Single-Institution Surgical Outcomes and Mortality Study.	2023	Mixed/other intracranial tumors	Retrospective clinical cohort/series	Single-center clinical cohort	2004–2021	Meningioma and GBM diagnosis-specific subgroups; age 80–89; n=45	Craniotomy/tumor resection; diagnosis-specific outcomes (meningioma vs GBM)	NR	None.
[71]	Transsphenoidal surgical treatment of pituitary adenomas in patients aged 80 years or older.	2014	Sellar/suprasellar tumors	Retrospective clinical cohort/series	Multicenter clinical cohort	1995–2012	Age ≥80; n=10; ASA II-III	Transsphenoidal/endoscopic pituitary-region surgery; no nonoperative comparator	mean follow-up 37.9 months	Grants-in-Aid for Scientific Research (Research on Hypothalamo-Pituitary Dysfunction), Ministry of Health, Labor and Welfare, Japan.

[72]	Endoscopic endonasal transsphenoidal surgery for patients aged over 80 years with pituitary adenomas: Surgical and follow-up results.	201 7	Sellar/suprasellar tumors	Retrospective clinical cohort/series	Single-center clinical cohort	2001–2014	Age ≥80; n=12	Transsphenoidal/endoscopic pituitary-region surgery; no nonoperative comparator	mean follow-up 34.4 months	Nil.
[73]	Risks and Benefits of Endoscopic Transsphenoidal Surgery for Nonfunctioning Pituitary Adenomas in Patients of the Ninth Decade.	201 7	Sellar/suprasellar tumors	Retrospective clinical cohort/series	Institutional clinical cohort; center count not stated	2007–2015	Age 80-85; n=15; comparator age 65-75 n=49	Endoscopic transsphenoidal surgery; age 80–85 vs age 65–75 comparator	mean follow-up 16.86 months	Not reported
[74]	Outcomes Following Transsphenoidal Pituitary Surgery in the Elderly: A Retrospective Single-Center Review.	201 9	Sellar/suprasellar tumors	Retrospective clinical cohort/series	Single-center clinical cohort	1995–2017	Age ≥80; n=21; comparator age 70-79 n=94	Transsphenoidal surgery; age ≥80 vs age 70–79 comparator	mean follow-up 27 months	Not reported
[75]	Exploring endocrinological pitfalls in pituitary surgery in the elderly.	202 3	Sellar/suprasellar tumors	Retrospective clinical cohort/series	Institutional clinical cohort; center count not stated	2006–2019	Age ≥75; n=29; ASA I-II only	Transsphenoidal/endoscopic pituitary-region surgery; no nonoperative comparator	NR	Japan Society for the Promotion of Science (JSPS) KAKENHI (JP22K09242).

[3]	Pituitary surgery outcome in patients 75 years and older: a retrospective study.	2023	Sellar/suprasellar tumors	Retrospective clinical cohort/series	Single-center clinical cohort	2008–2022	Age ≥75; n=155; median age 78	Transsphenoidal/endoscopic pituitary-region surgery; no nonoperative comparator	Median follow-up was 13 months (range: 3-96)	Not reported
[23]	Clinical Management Considerations for Craniopharyngioma in Geriatric Populations: An Assessment of the Surveillance, Epidemiology, and End Results Database.	2025	Sellar/suprasellar tumors	Retrospective database/registry analysis	SEER; Population cancer registry	2000–2022	SEER 2000-2022; age ≥80; n=74	No resection vs subtotal/gross-total resection; radiotherapy also assessed	NR	No external funding.
[24]	Surgical management in very elderly patients with pituitary adenoma: a multivariable assessment of the surveillance, epidemiology, and end results database.	2025	Sellar/suprasellar tumors	Retrospective database/registry analysis	SEER; Population cancer registry	2000–2022	SEER 2000-2022; age ≥85 n=686; age 75-84 n=2571	No resection vs subtotal/gross-total resection; radiotherapy also assessed	NR	No funding was utilized for the present study.
[4]	Spinal Meningioma Surgery in Octogenarians: Functional	2022	Spinal tumors	Retrospective clinical cohort/series	Single-center clinical cohort	2005–2020	Age ≥80; n=30	Posterior decompression and microsurgical resection; no nonoperative comparator	2-year follow-up	No external funding.

	Outcomes and Complications over a 2-Year Follow-Up Period.									
[76]	Clinical predictors of overall survival in very elderly patients with spinal osteosarcoma: an analysis of the surveillance, epidemiology, and end results database.	2026	Spinal tumors	Retrospective database/registry analysis	SEER; Population cancer registry	2000–2022	Age ≥75; n=117; grade-adjusted subgroup n=44	No resection vs STR vs GTR; chemotherapy and radiotherapy exposures also assessed	NR	No grant from funding agencies in the public, commercial, or not-for-profit sectors.
[9]	Surgical Intervention in Very Elderly Patients with Spinal Ependymoma: A National Cancer Database Analysis.	2026	Spinal tumors	Retrospective database/registry analysis	NCDB; National clinical/administrative database	NR	Age ≥75; n=126	Surgery vs no surgery (age ≥75 cohort; age 65–74 comparison cohort); radiotherapy also assessed	NR	No external funding.
[5]	Differential Impact of Advanced Age on Clinical Outcomes After Vestibular Schwannoma Resection in the	2021	Vestibular schwannoma	Retrospective clinical cohort/series	Mayo Clinic; single-center clinical cohort	1999–2020	Sporadic unilateral vestibular schwannoma; age ≥75; n=24, including 8 patients age ≥80	GTR vs near-total vs subtotal resection; no nonsurgical comparator	NR	No funding or financial support.

	Very Elderly: Cohort Study.									
[16]	Vestibular schwannoma surgery in the ninth decade of life: a case series.	2024	Vestibular schwannoma	Retrospective clinical cohort/series	Paris; single-center clinical cohort	2009–2020	Histologically confirmed vestibular schwannoma; age ≥80; n=13	Planned partial resection vs GTR; no nonsurgical comparator	Mean follow-up was 42.4 ± 44.7 months	None.
